# Potential progression biomarkers of diabetic kidney disease determined using comprehensive machine learning analysis of non-targeted metabolomics

**DOI:** 10.1038/s41598-022-20638-1

**Published:** 2022-09-29

**Authors:** Yosuke Hirakawa, Kentaro Yoshioka, Kensuke Kojima, Yasuho Yamashita, Takuma Shibahara, Takehiko Wada, Masaomi Nangaku, Reiko Inagi

**Affiliations:** 1 Division of Nephrology and Endocrinology, The University of Tokyo Graduate School of Medicine, Tokyo, Japan; 2 Kyowa Kirin Co., Ltd., Tokyo, Japan; 3 Division of Chronic Kidney Disease Pathophysiology, The University of Tokyo Graduate School of Medicine, Tokyo, Japan; 4 Research and Development Group, Hitachi, Ltd., Tokyo, Japan; 5 Division of Nephrology, Endocrinology and Metabolism, Tokai University School of Medicine, Isehara, Japan

**Keywords:** Kidney, Metabolomics

## Abstract

Diabetic kidney disease is the main cause of end-stage renal disease worldwide. The prediction of the clinical course of patients with diabetic kidney disease remains difficult, despite the identification of potential biomarkers; therefore, novel biomarkers are needed to predict the progression of the disease. We conducted non-targeted metabolomics using plasma and urine of patients with diabetic kidney disease whose estimated glomerular filtration rate was between 30 and 60 mL/min/1.73 m^2^. We analyzed how the estimated glomerular filtration rate changed over time (up to 30 months) to detect rapid decliners of kidney function. Conventional logistic analysis suggested that only one metabolite, urinary 1-methylpyridin-1-ium (NMP), was a promising biomarker. We then applied a deep learning method to identify potential biomarkers and physiological parameters to predict the progression of diabetic kidney disease in an explainable manner. We narrowed down 3388 variables to 50 using the deep learning method and conducted two regression models, piecewise linear and handcrafted linear regression, both of which examined the utility of biomarker combinations. Our analysis, based on the deep learning method, identified systolic blood pressure and urinary albumin-to-creatinine ratio, six identified metabolites, and three unidentified metabolites including urinary NMP, as potential biomarkers. This research suggests that the machine learning method can detect potential biomarkers that could otherwise escape identification using the conventional statistical method.

## Introduction

Diabetic kidney disease (DKD) remains the leading cause of end-stage kidney disease in developed and developing countries^1,2^. The poor predictability of progression rate for each patient with classical risk factors such as blood pressure and albuminuria indicates the difficulty in managing patients with DKD and conducting clinical trials^3^. Therefore, serum or urinary biomarkers for identifying rapid decliners of DKD have been the focus of intensive research. Early studies attempted to identify biomarkers of DKD progression using a pathophysiological pathway-based approach. These potential biomarkers include serum soluble tumor necrosis factor (TNF) alpha (TNFα), soluble TNF receptor 1 (sTNF-R1), and soluble TNF receptor 2 (sTNF-R2). These potential biomarkers were primarily targeted because of the well-known importance of the TNFα pathway in DKD. Recent progress in omics analysis, such as genomics, transcriptomics, and metabolomics, has allowed the application of multi-omics analysis for biomarker discovery in a non-biased manner. The omics approach is a powerful tool to discover novel disease pathways and unpredicted biomarkers, especially in kidney disease, because of the application of urine sample analysis^4–7^. In contrast, a large number of variables require strict statistical tests in the conventional approach to avoid false discovery, and potential biomarkers may be missed, especially in studies with small sample sizes. There are two types of metabolomics: targeted metabolomics and non-targeted metabolomics^8,9^. While targeted metabolomics measures defined substances identified from a priori knowledge, non-targeted metabolomics measures non-defined substances. This approach brings in substances that could be good biomarkers and substances with pathogenic roles. Because non-targeted metabolomics have yielded over 1000 measurement objects, we need to perform strict statistical tests, restricting the utility of omics analysis in small-cohort pilot studies. To increase the utility of pilot studies, which have important roles in exploring the potential of biomarkers or biomarker combinations to, therefore, determine the value and sample size of future scaled-up studies^10^, a breakthrough to detect biomarker candidates from numerous variables in a limited number of participants is eagerly awaited.

Machine learning is bringing innovation to data science, which is also applicable in medicine including nephrology^11^. However, an important problem for the application of machine learning to clinical studies is that ordinary machine learning cannot yield transparent and simple results. To implement the results of a clinical study in real-world medicine, physicians need sufficient justification of the results, with the need for informed consent or shared decision-making. Overfitting is another problem. Machine learning can result in a model with extremely high performance in the initial training data, but then loses its performance in the validation data. This overfitting is a result of deep and complicated model construction with limited data, thereby compromising the extrapolability of the model^12^. It is important to pay strict attention to overfitting when handling omics data in cohort studies because of the large number of variables in a relatively limited pool of patients. However, if these problems are resolved, machine learning will certainly be a useful tool for discovering biomarkers for diseases, even when analyzing small-cohort results. Moreover, because “statistically non-significant results do not ‘prove’ the null hypothesis” considering uncertainty^13^, it is important to identify potential biomarkers that are labeled as non-significant in conventional statistical tests.

We previously performed metabolomics of human and animal samples to identify biomarkers to predict the rapid progression of DKD^14^. In that study, we analyzed the identified metabolites using conventional statistical analysis, focused on lysophospholipids, and elucidated their pathological role. However, in the current study, we performed explainable machine learning analysis using non-targeted metabolomics of plasma and urine samples of patients with advanced DKD and identified new biomarkers, including unidentified metabolites, for rapid decliners while avoiding overfitting.

## Results

### Cohort formation and conventional analysis

We included 150 patients in the UT-DKD cohort and 135 patients completed the follow-up visit. Patients who ceased to participate in the study were 1 patient who withdrew consent, 3 with cancer, and 11 who were referred to other hospitals for personal reasons. The baseline characteristics of the 135 patients are summarized from a previous report and are shown in Supplementary Table 2^14^. We defined rapid decliners of DKD as patients whose annual estimated glomerular filtration rate (eGFR) change rate was below − 10% of baseline eGFR, which corresponded to the surrogate endpoint in chronic kidney disease, a %GFR change of less than − 30% over 2 or 3 years^15^. In the UT-DKD cohort, 14 patients were classified as rapid decliners. We next divided non-rapid decliners into three groups according to the eGFR change rate: patients whose eGFR change rate was above 0% as group1 (n = 46), below 0% and above − 3.3% as group2 (n = 34)^16^, below − 3.3% and above − 10%/year as group3 (n = 39), and < 10%/year (rapid decliner) as group4 (n = 14).

Next, relative MS area of the baseline plasma and urinary metabolite of each group were compared between the rapid decliners and other participants. The urinary and plasma metabolites with good predictive values are shown in Table 1 and Supplementary Table 3.

**Table 1:**
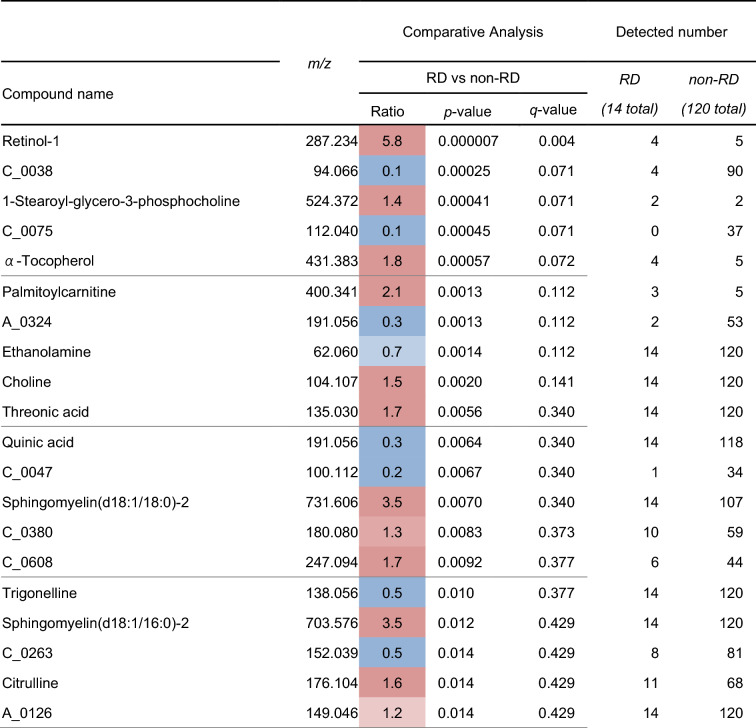
Logistic analysis for rapid decliner using urinary metabolome.

*RD* rapid decliners.

**Figure 1:**
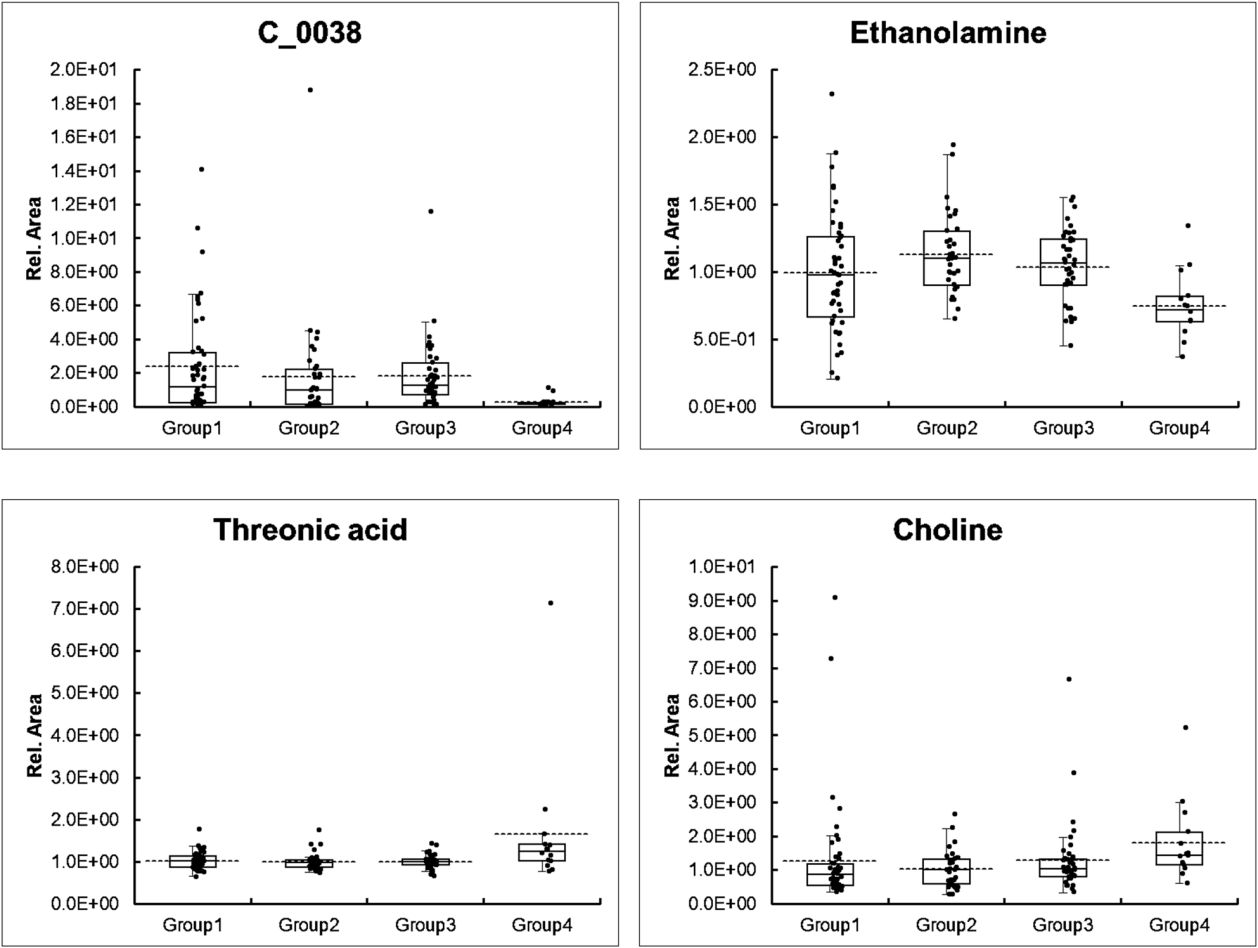
Representative relative MS area of promising metabolites. A point plot and box plot of four promising metabolites, C_0038, ethanolamine, threonic acid, and choline, are shown. The vertical bar shows the relative area of each metabolite. The dotted line is equal to the average. Annual eGFR change rate for each group was as follows; group1, above 0%; group 2, below 0% and above − 3.3%; group3, below − 3.3% and above − 10%, and group 4, below -10%.

**Figure 2:**
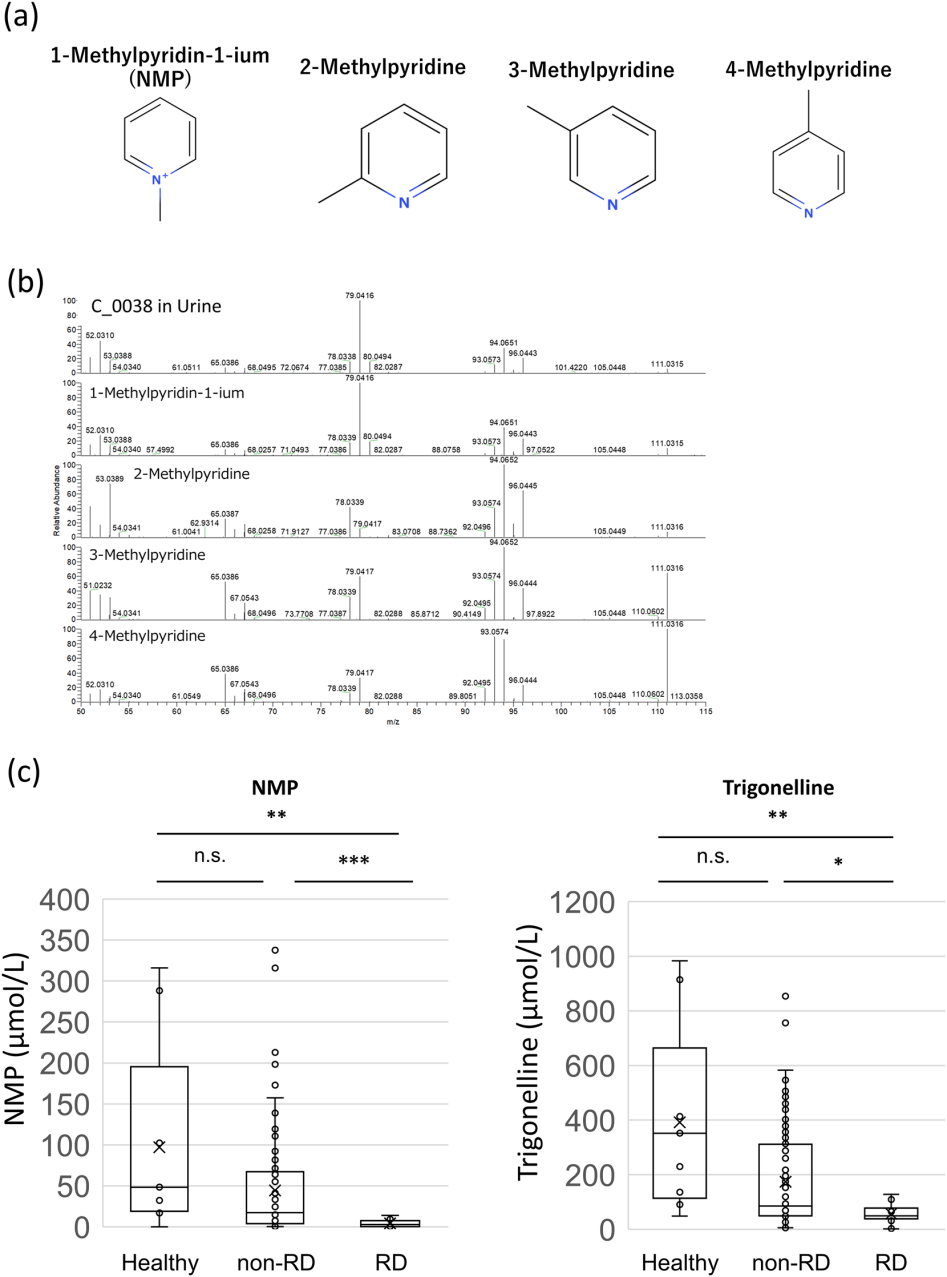
Identification of C_0038 as NMP, and concentration of NMP and trigonelline. (**a**) Four predicted structural formula of C_0038. (**b**) Identification of C_0038 as NMP. Result of MS/MS analysis of C_0038 in the representative human urine analyzed in this research and standard chemicals of four possible structural formula was shown (Collision Energy: 30 eV). A similar pattern was detected in the C_0038 and 1-methylpyrydin-1-ium (NMP). Human healthy urine samples were obtained from BioIVT (U.S.) (**c**) Urinary concentration of NMP and trigonelline. Urinary concentration of NMP (upper) and trigonelline (lower) was measured in healthy subjects, patients with diabetic kidney disease who were not rapid decliners, and rapid decliners among diabetic kidney disease patients. Data indicated by Tukey box plot. *p < 0.05, **p < 0.01, ***p < 0.005, n.s. not significant (Kruskal–Wallis test). *RD* rapid decliner.

Several metabolites with good predictive values, such as urinary retinol-1, were detected in less than half of the participants in each group. Considering its clinical applications, easily detectable biomarkers are most appropriate; therefore, we focused on metabolites detected in at least half of all participants. Several urinary metabolites had good predictive values, and the relative MS area of representative urinary metabolites are shown in Fig. 1. A non-defined substance, urinary C_0038, seemed to be the best predictor of those metabolites examined because it had a good predictive value and was detected in approximately 70% of the participants. Therefore, we predicted the structure of C_0038 and confirmed the structure by mass spectrometry. These analyses led to the identification of this metabolite as 1-methyloyridin-1-ium (NMP) (Fig. 2a and b, and Supplementary Figure 1). It was also confirmed that the urinary NMP concentration was lower in rapid decliners than in non-rapid decliners of DKD and healthy subjects (Fig. 2c). Trigonelline, a metabolite that has been measured in this metabolomics study, is a precursor of NMP, and its concentration was also lower in rapid decliners, similar to NMP (Fig. 2c). These results indicate that urinary NMP and trigonelline can be used as markers for predicting the progression of DKD.

### Biomarker candidates based on decliner prediction models

Next, we applied a machine learning approach to identify potential biomarkers using data from non-targeted metabolomics. First, we examined whether the combination of clinical data and metabolomics data would improve prediction performance. We compared the prediction performance of three datasets: metabolomic dataset, clinical dataset, and metabolomic and clinical dataset. Table 2 summarizes the prediction performance of the results for rapid decliners. Other conventional prediction performances (F-measure, Accuracy, Precision, Recall, false positive rate, and false negative rate,) are represented in Supplementary Table 4.

**Table 2:** Comparison of four analysis methods.

	Training			Test		
	Metabolomic dataset	Clinical dataset	Metabolomic and clinical dataset	Metabolomic dataset	Clinical dataset	Metabolomic and clinical dataset
Deep learning	0.758 ± 0.028	0.766 ± 0.026	0.770 ± 0.035	0.688 ± 0.225	0.651 ± 0.288	0.775 ± 0.182
Logistic regression	0.739 ± 0.052	0.633 ± 0.064	0.754 ± 0.046	0.597 ± 0.214	0.557 ± 0.179	0.596 ± 0.222
Random forest	0.751 ± 0.037	0.784 ± 0.04	0.768 ± 0.028	0.575 ± 0.318	0.644 ± 0.177	0.665 ± 0.272
SVM	0.704 ± 0.039	0.772 ± 0.061	0.738 ± 0.047	0.522 ± 0.217	0.611 ± 0.263	0.643 ± 0.252

The 12 results were combinations of 4 models (deep learning, logistic regression, random forest, and support vector machine (SVM) and three datasets, respectively. The AUCs of models were calculated using tenfold double cross validation (tenfold DCV)^17^. In tenfold DCV, the samples are split randomly into 10 folds and the test set as well as the training set are defined for each fold. A model was constructed using the training set and the trained model was evaluated using the test set for each fold, i.e., 10 times in total. The respective sample sizes for the training and test sets are 121 and 14 in five-folds and 122 and 13 in the other five folds. The prediction performance for each learning model had a mean AUC value over the 10 folds. The AUC value of the test set was highest in metabolomic and clinical dataset with the deep learning model, suggesting that a combination of clinical and metabolomics data would be useful. Ignoring the statistical error that inevitably becomes large for the test set because of the small sample size, the mean AUC value of the test sets (0.775 ± 0.182) is comparable to that of the training sets (0.770 ± 0.035), which suggests that the deep learning model seemed to avoid overfitting for metabolomic and clinical dataset. Supplementary Table 6 summarizes the list of features and their importance scores generated from the deep learning model for metabolomic and clinical dataset, the one with the best AUC value (0.775 ± 0.182). The importance score of each table evaluates the extent to which each feature contributes to raising (or lowering) the model’s output probability to classify rapid decliners. The importance scores were sorted in descending order, and we truncated the features whose absolute values were < 0.04 in the list. For example, a high urinary threonic acid signal increased the rapid decliner probability, while the negative missing flag of urinary NMP lowered the rapid decliner probability. We selected 50 features according to the following criteria: 39 features whose absolute importance score was over 0.25 and 11 features that were known to be related to the pathogenesis or progression of DKD or related to NMP metabolism (Supplementary Tables 7 and 8)^14,18–20^. Among the 50 features, 30 were continuous variables and 20 were binary variables (missing flag). Notably, only two clinical parameters, systolic blood pressure and urinary albumin-to-creatinine ratio, were included. Of the remaining 48 parameters, 14 were known plasma metabolites and 7 were unidentified plasma metabolites, 16 were known urinary metabolites, and 11 were unidentified urinary metabolites.

### Piecewise linear model and handcrafted linear regression model

**Figure 3:**
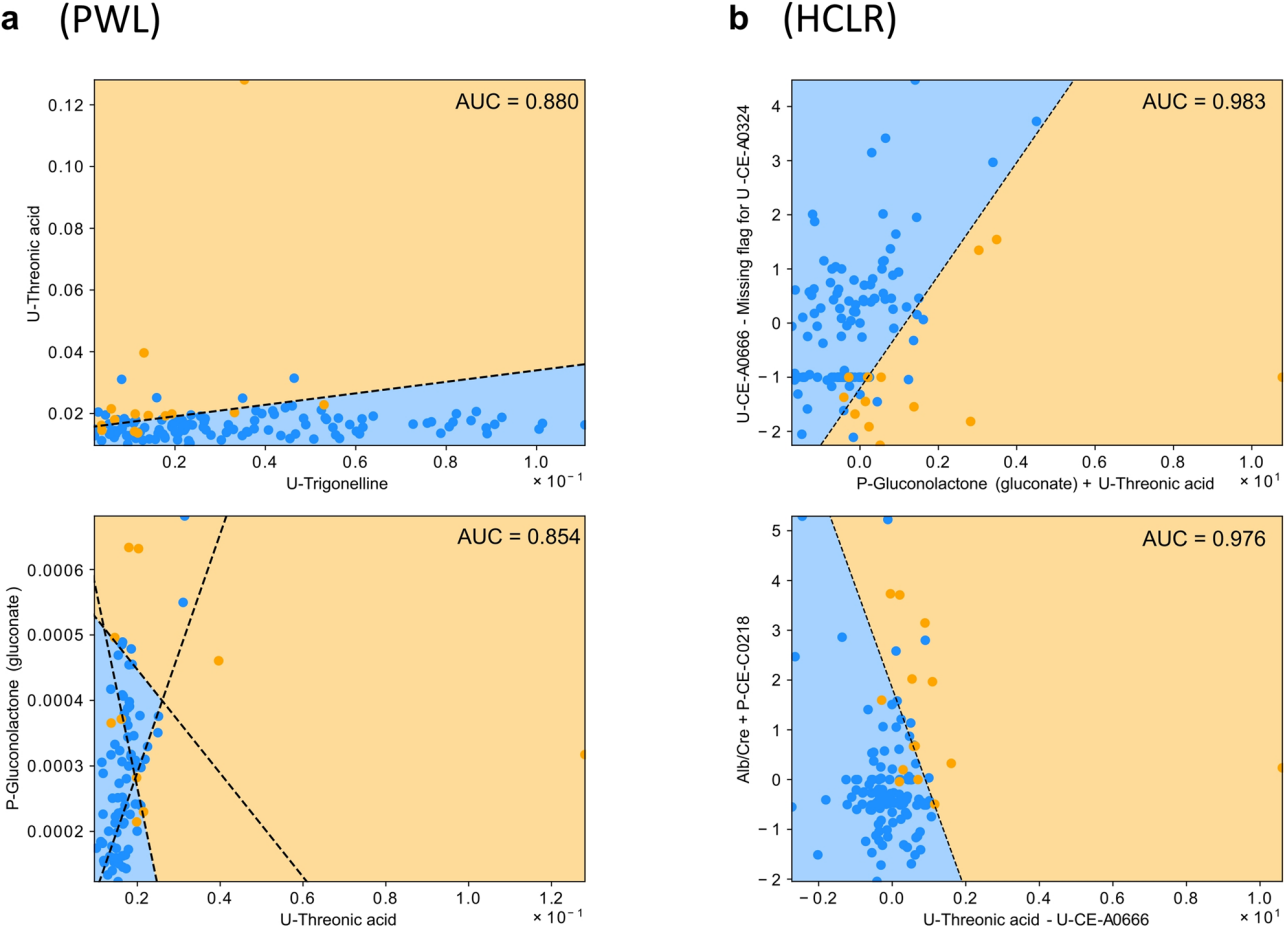
Representative plots of PWL model and HCLR model. Representative plots of (**a**) PWL and (**b**) HCLR models with high AUC values. The orange and blue areas show the regions classified as rapid decliners and others by each model, respectively. The orange and blue points correspond to the rapid decliners and others in the dataset, respectively. The dashed line represents the classification boundaries by setting the threshold level of the rapid decliner probability to the one with which the f1-measure was maximum. *PWL* piecewise linear, *HCLR* handcrafted linear regression.

Targeting these 50 features, we investigated the utility of PWL and HCLR models. For the PWL model, the missing flag cannot be used as a binary parameter. Therefore, 30 features were employed in the PWL model, whereas 50 features were employed in the HCLR model. Representative classifications that displayed the highest and second-highest AUC in each model are shown in Fig. 3. We defined high-AUC models by 65 PWL models with AUC values over 0.8, 23,040 HCLR models with AUC values over 0.9, and features frequently included in high-AUC models as candidates of biomarkers for rapid decliner prediction (Table 3, Supplementary data 1 and 2).

**Table 3:** Representative features in PWL models and HCLR models.

Factor	Frequency	Mean	Difference from overall mean	Manually selected features
**PWL**				
P-CE-C0218	17	0.801	0.080	No
Systolic_blood_pressure	10	0.763	0.042	No
P-Guanidinosuccinic acid	8	0.773	0.052	Yes
U-Threonic acid	8	0.761	0.041	No
P-CE-A0242	8	0.746	0.025	No
U-Xanthine	8	0.769	0.048	Yes
P-Kynurenine	7	0.763	0.042	Yes
Alb/Cre	6	0.756	0.035	No
U-1-Palmitoyl-glycero-3-phosphocholine	6	0.734	0.013	Yes
U-Xanthosine	6	0.741	0.020	No
U-Sphingomyelin(d18:1/16:0)	5	0.739	0.018	Yes
U-CE-C0134	5	0.763	0.042	No
P-Homocitrulline	4	0.758	0.037	No
P-Gluconolactone (gluconate)	4	0.751	0.030	Yes
P-FA(17:0)-2 Heptadecanoic acid-2	4	0.743	0.022	No
**HCLR**				
U-Threonic acid	15,074	0.897	0.050	No
P-CE-C0218	7183	0.884	0.037	No
Missing flag for U-CE-A0324	5766	0.873	0.026	No
U-CE-A0666	4294	0.859	0.012	No
Alb/Cre	4171	0.874	0.027	No
U-Sphingomyelin(d18:1/16:0)	3739	0.873	0.026	Yes
Missing flag for U-CE-A0401	2749	0.850	0.003	No
Missing flag for U-NMP	2718	0.868	0.021	No
P-Gluconolactone (gluconate)	2636	0.858	0.011	Yes
Missing flag for U-Dehydroisoandrosterone 3-sulfate-2	2286	0.858	0.011	No
U-1-Palmitoyl-glycero-3-phosphocholine	2217	0.862	0.015	Yes
Missing flag for U-U-CE-C0352	2141	0.856	0.009	No
Missing flag for P-XC0138	2075	0.853	0.007	No
Missing flag for P-Solanidine	1942	0.856	0.009	No
U-Trigonelline	1867	0.857	0.011	Yes
Missing flag for P-FA(15:1)-1–2 FA(15:1)-2–2	1858	0.854	0.007	No
P-Kynurenine	1772	0.856	0.010	Yes
Missing flag for U-CE-C0047	1770	0.855	0.009	No
P-CE-A0242	1727	0.854	0.007	No
Systolic_blood_pressure	1659	0.856	0.009	No

**Figure 4:**
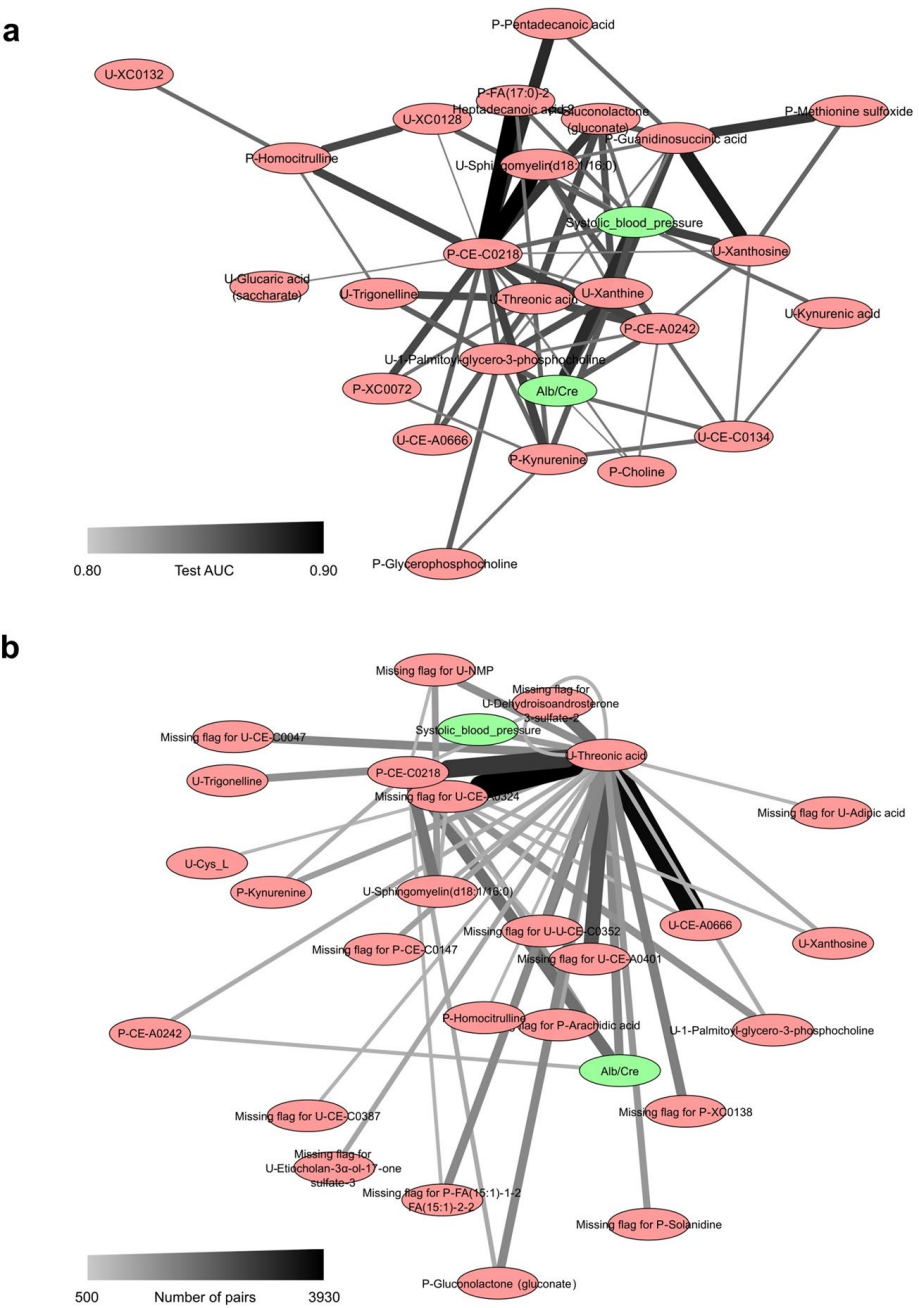
Graph network of features in PWL models and HCLR models. Graph network derived from high-AUC models of (**a**) PWL and (**b**) HCLR. The width of an edge between two nodes represents the interaction strength between the biomarker candidate’s pair that the two nodes represent. For visibility, we ignored all nodes whose interaction strength was smaller than a threshold value: 0.8 in PWL models and 500 in HCLR model. *PWL* piecewise linear, *HCLR* handcrafted linear regression.

We also supposed that the prognostic potential of each feature could be assessed by the difference between the average AUC of the equation including the feature and the overall average (Table 3). In HCLR models, however, it is not fair to examine the mean of all models that contain each feature because the presence of a high-accuracy HCLR model often leads to the presence of a low-accuracy HCLR model; for example, A–B is a good biomarker, but A + B becomes a poor biomarker because the substitution of B is essential. Therefore, we showed the average of the top 10% of AUCs of equations including the feature (instead of the average of all the AUCs of equations including the feature) in the HCLR models. Two clinical features, systolic blood pressure and urinary albumin-to-creatinine ratio; five identified metabolites, plasma kynurenine, plasma gluconolactone (gluconate), urinary threonic acid, urinary 1-palmitoyl-glycero-3-phosphocholine, and urinary sphingomyelin(d18:1/16:0); and two unidentified metabolites, plasma CE-C0218 and plasma CE-A0242, were identified in both PWL and HCLR models. These features are considered potential biomarkers. Twenty-one binary features (missing flags) were only included in the HCRL model, and the missing flags for U-CE-A0324, U-NMP, and U-Dehydroisoandrosterone 3-sulfate-2 seemed to be potential biomarkers considering the average AUC. The combination of multiple features is important; therefore, the combination frequencies of the high-AUC models are shown in Fig. 4 (please see supplementary data 3 and 4 for full list). From this graph network, P-CE-C0218 and U-threonic acid respectively locate the center of the PWL and HCLR models’ networks, i.e., they connect with many other features. Also, C0218 and U-threonic acid have the strongest interactions among the interactions between all the feature pairs in the PWL and HCLR models, respectively. Therefore, we considered that P-CE-C0218 in the PWL model and U-threonic acid in the HCLR model appeared to be key features in creating high-AUC regression models.

## Discussion

In this study, we performed an explainable machine learning analysis using data from non-targeted metabolomics of the plasma and urine of patients with DKD. We also analyzed unidentified metabolites, and the number of variables was as high as 3388. A large number of variables hindered us from identifying potential biomarkers; therefore, we applied an explainable machine learning method to detect potential biomarkers. To the best of our knowledge, this is the first longitudinal study to focus on the results of non-targeted metabolomics in advanced DKD.

In this study, the conventional statistical test revealed that only urinary NMP could be a prognostic marker, but machine learning analysis revealed five identified metabolites and two unidentified metabolites that were listed in both HCLR and PWL models. A missing flag was set if the detection ratio of the feature value was more important than the measured value. Note that the missing flag is a binary feature; therefore, such features were only assessed using the HCLR model. Three missing flags, including the missing flag for urinary NMP, were likely potential biomarkers based on the results of the PWL models. Among these potential biomarkers, P-CE-C0218 and urinary threonic acid have good utility in combination with other biomarkers. This dissociation of the results stemmed from the difference between the univariate analysis and bi- or multivariate analysis. Using PWL or HCLR models, the prognostic utility of feature combinations can be assessed. For example, plasma CE-C0218 did not appear to be a good prognostic marker in the univariate analysis but turned out to be a promising prognostic marker when combined with other features (Supplementary Table 3 and Fig. 4).

In this study, we employ a two-step strategy to obtain explainable results. First, we selected 50 features using a deep learning method. This method, based on the deep learning model, was not expected to yield a result that was overfitted to the training data. The deep learning model in this study used a unified architecture characterized by the binding of each network layer and neurons in a mesh-like form^21^. This mesh-like form was designed to avoid the overfitting drawbacks of deep learning. It was found that the deep learning process yielded the highest AUC value in the test dataset, even though the metabolites with low detection frequency were not excluded (Table 2). Increasing the number of features could result in overfitting; in other words, including more variables could result in a lower AUC value in the test set. In the deep learning models, the difference between the trained AUC value and test AUC value for metabolomic and clinical dataset was apparently smaller than those for metabolomic dataset and clinical dataset, unlike the other machine learning models. Our deep learning model avoids overfitting metabolomic and clinical dataset with larger features. The second step was comparing the selected 50 features using PWL and HCLR models. This two-step strategy enabled us to avoid overfitting and yielded explainable results.

To date, intensive research has focused on the identification of biomarkers to predict the progression of DKD. The urinary albumin-creatinine ratio is a classical ratio, and urinary sTNFR1, sTNFR2, and KIM1 are representative of new biomarkers. One study of early and advanced DKD cohorts indicated that the AUC value of the prognostic model for renal endpoint was 0.680 in clinical models alone, ranging from 0.709 to 0.735 in clinical models plus one biomarker (either urinary sTNFR1, TNFR2, or KIM1) and 0.752 in clinical models plus all three biomarkers^22^. In another recent study that applied a random forest model to patients with DKD, the AUC value for the renal endpoint was 0.61 in the validation set using the clinical model only and 0.77 in the validation set using the clinical model plus urinary sTNFR1, sTNFR2, and KIM-1^23^. These results indicate that the clinical model could not precisely predict the renal outcome. However, considering an AUC value of approximately 0.75, the prediction model using clinical parameters and known biomarkers remains insufficient for clinical use. Therefore, novel biomarkers for DKD are still needed. Therefore, we focused on metabolomics, especially non-targeted comprehensive metabolomics, in advanced DKD.

Research highlighting the importance of metabolomics as a biomarker for DKD is limited. In a longitudinal study, a recent report that examined 13 metabolites in the Chronic Renal Insufficiency Cohort study cohort demonstrated the prognostic value of 3-hydroxyisobutyrate and 3-methylcrotonyglycine, citric acid and aconitic acid^7^. Another study examined urinary metabolites in patients with type 1 diabetes using NMR and advocated that several urinary metabolites such as leucine, isoleucine, and threonine can be biomarkers for DKD progression^24^. These studies examined a limited number of metabolites (< 100); therefore, the threshold *p* value for traditional statistical tests was relatively loose. Our research employed non-targeted, comprehensive metabolomics; therefore, we identified 474 plasma metabolites, 442 urinary metabolites, and other unidentified metabolites and examined their utility as biomarkers in DKD progression. Metabolites with low detection frequency, were not excluded, as these specific metabolites could be potential biomarkers and rapid decliners represented only 10.4% of the patients. In light of the low proportion of rapid decliners, it was deduced that metabolites with low detection frequency could also be biomarkers. This approach did not limit the number of variables, however statistical tests were more rigorous. If we used only a traditional statistical test, we could not detect any metabolites under the threshold q value of 0.05. Such strict statistical tests may veil the potential biomarkers. In this study, we attempted a novel analysis approach that can widely detect potential metabolites by employing machine learning.

In this study, we focused on machine learning methods that maximize the utilization of the discovery cohort, that is, a large number of variables in a limited number of patients. Although we performed double cross-validation as a strict internal validation, we did not perform an external validation study. Therefore, the detected metabolites were not externally validated. Nevertheless, two metabolites, urinary threonic acid and plasma CE-C-0218, appeared to be good predictors of rapid decliners of DKD, because they were listed higher in both the PWL and HCLR when compared with urinary alb/Cre, a known prognostic marker in DKD. However, the relationship between threonic acid and kidney disease or diabetes has rarely been reported. A recent report indicated that urinary threonic acid is a potential biomarker for monitoring nonsteroidal anti-inflammatory drug use in cats^25^. However, no previous report has indicated its significance in human kidney diseases. CE-C-0218 was an unidentified metabolite. This metabolite is 4-(trimethylammonio) but-2-enoate. We should wait for future research and further meta-studies that perform non-targeted metabolomics in DKD patients to judge whether this metabolite is associated with DKD progression and whether this metabolite can be a good predictive marker.

The limitations of this study are as follows. First, sample size was as small as 14 rapid decliners in a total of 135 patients. Second, we analyzed only the discovery cohort. Metabolites highlighted in this study, such as threonic acid and CE-C-0218, were not considered biomarkers in this study. This study focused on the extraction of potential biomarkers from the screening cohort, and we did not include a validation cohort. Thus, the possibility of overfitting remained. Third, in this study, we did not set hard outcomes, such as dialysis induction and overall death. During this short 30-month research period, no patient developed end-stage kidney disease; therefore, we set a surrogate endpoint. An eGFR change of − 10%/year corresponds to − 20%/2 years, which has been recently advocated as a good surrogate endpoint^15^. Fourth, although we used a non-target metabolomic approach and AUC-based potential metabolite identification, several metabolites were manually selected according to known biological significance (Supplementary Table 7). Although two metabolites, urinary threonic acid and plasma CE-C0218, were selected according to the importance score or, in other words, not manually selected, the results must be interpreted with caution.

In summary, we performed non-targeted metabolomics and comprehensive machine learning analysis in patients with DKD and found that machine learning analysis can reveal features that are important in DKD progression prediction. This technique can be applied to other discovery studies and will be helpful for researchers to maximize the utilization of discovery cohort studies.

## Methods

### Cohort formation and sample collection

This study was approved by the ethics committee of the University of Tokyo Graduate School of Medicine (ethical approval number 10660). The UT-DKD cohort consisted of CKD G3 DKD patients^14^. The inclusion criteria were diabetic CKD G3 patients over 20 years of age who were not previously diagnosed with other kidney diseases, such as glomerulonephritis and polycystic kidney disease. The exclusion criteria were as follows: systolic blood pressure > 170 mmHg, HbA1c > 9.5%, any form of cancer, kidney diseases other than DKD or nephrosclerosis, organ transplant as a recipient, and those who had undergone systemic steroid therapy within 1 month before enrollment.

A total of 150 patients were recruited between January 2015 and September 2016. Written informed consent was obtained from all participants. Plasma and urine samples were collected at the baseline and follow-up visits (set 10 months after the baseline visit). All plasma and urine samples were collected under fasting conditions, defined as at least 10 h of fasting, and were preserved at − 80 °C. Information about family history, medical history, smoking and drinking habits, and medication were collected at the baseline visit. Laboratory data were collected from electrical medical record (collected items are shown in Supplementary Table 1). The Japanese-MDRD equation was used to calculate eGFR^26^, and all eGFR data for 30 months from the baseline visit were collected. The annual decline rate of eGFR was calculated at every 10 months (i.e., at four time points) using the least squares method.

### Sample preparation and mass spectrometry (MS) analysis for metabolomic profiling

Metabolomic analyses were performed by Human Metabolome Technologies Inc. (HMT, Tokyo, Japan). Plasma and urine samples were analyzed by capillary electrophoresis time-of-flight MS (CE-TOF–MS) and liquid chromatography time-of-flight MS (LC-TOF–MS) using HMT Advanced Scan methods^27^. All samples at baseline and 10 months were measured once independently.

For CE-TOF–MS analysis, 50 µL plasma samples were added to 450 µL methanol containing internal standards (HMT). The solution was mixed with 500 µL chloroform and 200 µL water and then centrifuged at 2300×*g* for 5 min at 4 °C. The upper layer was centrifugally filtered through a 5-kDa cut-off filter (HMT; Ultrafree MC-PLHCC) at 9100×*g* for 120 min at 4 °C and reconstituted in water. Next, 20 µL urine was added to 80 µL of water containing internal standards. This solution was centrifugally filtered through a 5-kDa cut-off filter at 9100×*g* for 60 min at 4 °C.

For LC-TOF–MS analysis, 500 µL of plasma samples were added to 1.5 mL acetonitrile with 1% formic acid containing an internal standard solution. The solution was centrifuged at 2300×*g* for 5 min at 4 °C, and the supernatant was filtered using a hybrid SPE phospholipid cartridge (55261-U; Sigma-Aldrich, St. Louis, MO, USA). After drying, the precipitate was reconstituted in 50% (v/v) isopropanol. Urine samples (100 µL) were mixed with 300 µL methanol containing internal standards and centrifuged at 2300×*g* for 5 min at 4 °C. The supernatant was dried and reconstituted in 50% (v/v) isopropanol. Metabolites were measured by CE-TOF–MS and LC-TOF–MS using HMT Advanced Scan methods, as previously described^27^. For relative quantification, each MS peak intensity was normalized based on the sample volumes and internal standards. For urine samples, MS intensity was normalized by the intensity of the creatinine peak. The annotation of each metabolite peak was identified using the HMT metabolite database^27^. For the quantitative comparison of each metabolite, the missing value was imputed with half of the minimum MS intensity in all detected subjects, as previously reported^28^. The annotation for unidentified metabolites is based on the m/z values of the target metabolite, which is estimated by referring to the KEGG* compound database. (*KEGG; Kyoto Encyclopedia of Genes and Genomes, https://www.genome.jp/kegg/compound/).

### Identification of C_0038 as 1-methylpyrydin-1-ium (NMP) using CE-MS/MS

A non-defined substance, urinary C_0038, was identified by CE-MS/MS-based substance structure estimation. First, the estimated molecular formula of C_0038 was calculated based on the results of exact MS spectrum and the isotope peak of CE-TOF–MS, which is described above as metabolomic analysis (estimated molecular formula: C6H6N−, C6H7N, C6H8N+). Next, we performed additional analysis using CE-MS/MS. The condition is as follows for CE, Capillary: Fused silica capillary i.d. 50 µm × 80 cm, Instrument: Agilent CE system, Run buffer: 1 M Formic acid, Voltage: 30 kV; for MS, Instrument: Thermo Q-Exactive plus, Polarity: Positive, Resolution: 140,000, Scan range 60–900 m/z. The MS/MS actual measurements and their retention times under 40 eV collision conditions were matched with in silico predictions using a metabolomics-based chemoinformatics approach reported previously^29^. Four candidates were estimated, and all of their commercial substances were matched to peaks in the urine sample using MS/MS. Finally, C_0038 was identified as NMP.

### Biomarker candidates based on decliner prediction models

First, we constructed prediction models to classify the patients into rapid decliners and non-rapid decliners and to extract important features (i.e., features that highly contribute to the classification) as biomarker candidates. Metabolomic dataset included baseline metabolomic parameters, clinical dataset included baseline clinical data, and metabolomic and clinical dataset included both clinical parameters and metabolomic data.

The variables featured in the three datasets were classified into binary (e.g., sex, family history of diabetes), multi-categorical (e.g., NIT, UBG), and quantitative variables (e.g., body height and hemoglobin). We set binary feature variables to 1 or − 1 values. Next, we performed one-hot encoding for multi-categorical variables; for one multi-categorical variable of K categories, we converted the variable into K variables, each of which takes 1 as the value if the sample belongs to the corresponding category and -1 otherwise. If a value in the binary and multi-categorical variables was missing, missing values were set to 0. The normalization process for each continuous quantitative variable was performed by subtracting the feature mean value and dividing by the standard deviation. For each ordinal quantitative variable, such as the frequency of drinking, we defined the normalized ordinal variables so that the values were expressed on a scale in a numerical order. Furthermore, we added a missing flag variable to each quantitative variable in the metabolomic data. Missing flag variables were created using the following process. There were two types of missing data for the quantitative variables. In the type-1 case, the value was smaller than the measured sensitivity. The values for the type-2 case were not measured. The missing flag variables had three states: 1 for the type-1 case, 0 for the type-2 case, and − 1 for not missing. If a value in the quantitative variables was missing, we set 0 as the missing value for the normalized feature variables. In this study, we replaced the missing values with 0 because the 0-value input did not change the output in the weighted-sum layers of the deep learning model. Thus, the 0-value input does not change the inference of the deep learning model. The numbers of feature variables in three datasets were 3311, 77, and 3388, respectively. All feature variables are summarized in Supplementary Table 5.

There are four prediction methods and three datasets (metabolomics dataset, clinical dataset, and metabolomic and clinical dataset) included in the prediction models. One of the prediction methods was a deep learning-based method using a point-wise linear (hereinafter referred to as deep learning) model^30^ (implemented using PyTorch 1.5.1, Python 3.7.4). This deep learning model derived the output value as a weighted sum of the input features whereby weights were calculated using a deep neural network. One can compute the importance of each feature using its weight value. Furthermore, the deep learning model used deep unified networks^21^ in which the network layers and neurons are connected in a mesh-like form. This mesh-like structure reduces the risk of overfitting. The other three methods, (1) logistic regression, (2) random forest, and (3) support vector machine (SVM), were adopted to build the baseline models (implemented using scikit-learn v0.21.3, Python 3.7.4) to validate the prediction performance of the deep learning models. The model output is the probability of a sample being classified as a rapid decliner. We calculated each model’s prediction performance using the area under the curve value evaluated by tenfold DCV^17^. We chose the best prediction performance model among the three deep learning models for three datasets and evaluated the importance score for each feature using the relative score^30^. We selected features as biomarker candidates by imposing importance scores greater than 0.25. The importance score of 0.25 indicates that among either rapid-decliner samples or non-rapid-decliner samples at least half of the samples considered that the feature is one of the top 10% of important features. We added manual selection of metabolomes with importance scores larger than 0.06, which were reported to be important in the pathogenesis or progression of DKD. Here, we need to restrict the number of features around 50 in the following analysis which is computationally expensive: the total number of models to be examined in one of the following analyses is 1.97 × 10^7^ for 50 features and 4.12 × 10^14^ for the whole 3388 features. We selected the features using the importance scores, since the features with higher importance scores definitely contribute to the prediction result individually. However, the importance score is not the perfect scoring method to measure the "importance" that humans often consider. For example, the importance score can underestimate the features that need to work with other features to affect the prediction. Therefore, we supplemented the features selected by the imperfect scoring method with the ones manually curated based on our biological knowledge.

### Relationships between biomarker candidates

Although we obtained the biomarker candidates and their importance scores based on the deep learning model that considers nonlinear interactions between the features, we could not identify how the features related to each other because of their complexity. Therefore, we investigated the explicit nonlinear relationship among biomarker candidates by constructing simple and comprehensive nonlinear models that classify rapid decliners with biomarker candidates using two methods. One method was to construct two-dimensional classification models (implemented by PyTorch 1.5.1, Python 3.7.4) that consist of 2–4 boundary lines derived by a piecewise linear function^31^, which we refer to as piecewise linear (PWL) models. The other method was to construct two-dimensional logistic regression models (implemented by scikit-learn v0.24.2, Python 3.7.4) with handcrafted feature vectors, which we refer to as handcrafted logistic regression (HCLR) models. The handcrafted feature vectors were calculated using all possible combinations of the four basic arithmetic operations (+, −, ×, and ÷) of the two biomarker candidates.

We evaluated the AUC values for all possible PWL and HCLR models. We then visualized the classification boundaries by setting the threshold level of the rapid decliner probability to the one where the f1-measure was maximum. The threshold level was well balanced between the true positive and false positive ratios. To investigate which features and feature combinations are often adopted in these simple models, we counted the number of models containing each single feature in the high-AUC models for each method, which we call each feature’s single frequency. In this study, we defined the high-AUC models using 65 PWL models whose AUCs were higher than 0.8 and 23,040 HCLR models whose AUCs were higher than 0.9. Additionally, we constructed a graph network to visualize the interactions between the biomarker candidates for each method. To evaluate the strength of the interaction between each feature pair in the HCLR models, we counted the number of models containing each feature pair in the high-AUC models. For PWL models, on the other hand, we define the interaction strength between each feature pair by the AUC of the PWL model of the feature pair because the PWL model that contains each feature pair is uniquely determined. In graph networks, the nodes and the width of the edges between two nodes represent the biomarker candidates and the interaction strength of the corresponding biomarker pair, respectively.

### Statistical analysis

The relative MS area of each metabolite in the plasma or urine was compared between the groups using the Mann–Whitney U test. Thereafter, the q value was calculated using the Benjamini–Hochberg method. Statistical significance was set at a *p* value of < 0.05.

### Ethical declarations

This study was approved by the ethics committee of the University of Tokyo Graduate School of Medicine (ethical approval number 10660) and performed in accordance with the Declaration of Helsinki and the institutional guidelines.

## Supplementary Information

Supplementary Information 1.

Supplementary Information 2.

Supplementary Information 3.

Supplementary Information 4.

Supplementary Information 5.

## Data Availability

The datasets used and analyzed during the current study available from the corresponding author on reasonable request.
