# Supplementary material for: Potential progression biomarkers of diabetic kidney disease determined using comprehensive machine learning analysis of non-targeted metabolomics
Source: Sci Rep. 2022 Sep 29;12:16287. doi: 10.1038/s41598-022-20638-1 (PMC9523033; doi:10.1038/s41598-022-20638-1)
Supplement: Supplementary file 1 — Supplementary Information 1. [file 41598_2022_20638_MOESM1_ESM.pptx]

## Slide 1
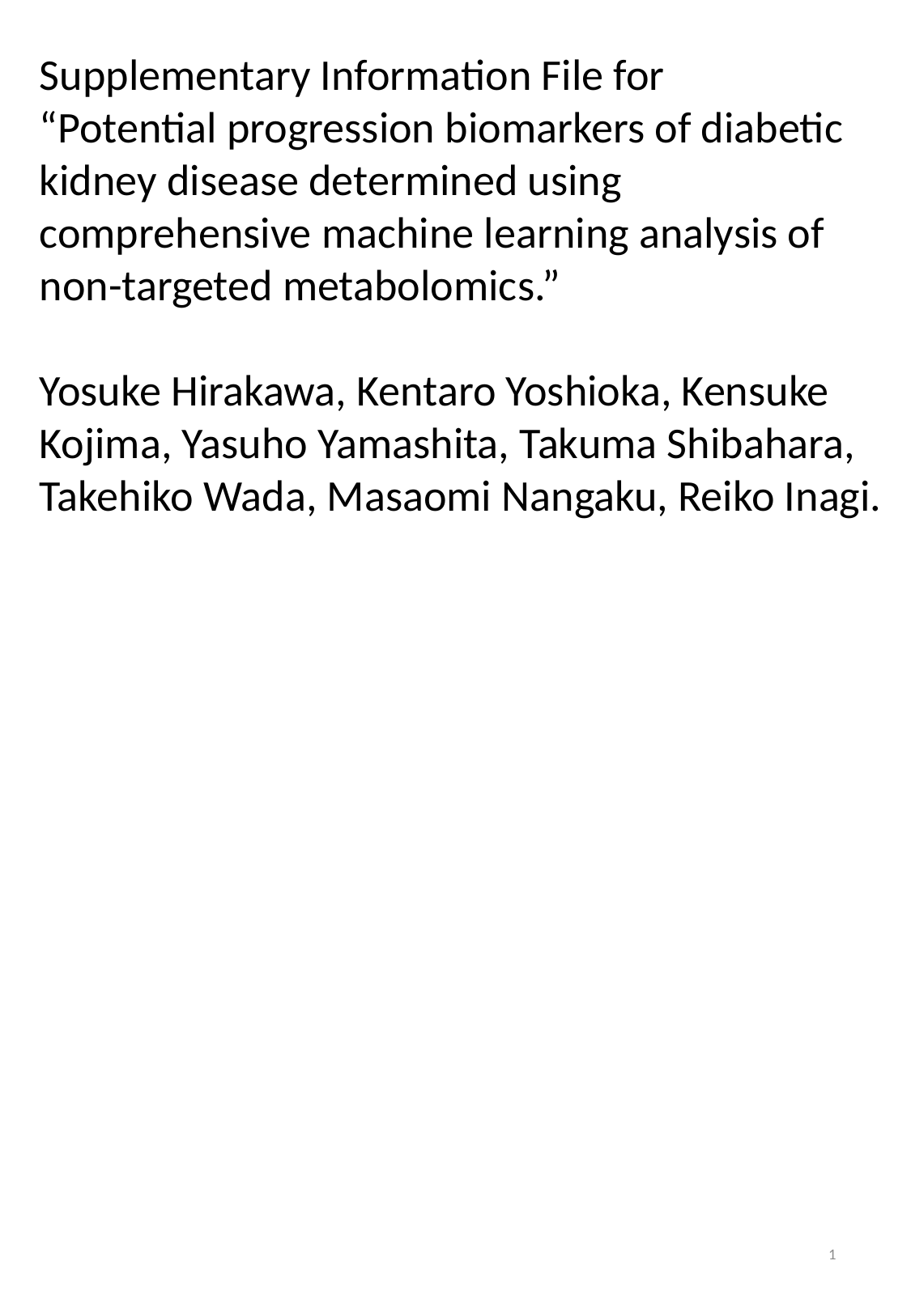

Supplementary Information File for
“Potential progression biomarkers of diabetic kidney disease determined using comprehensive machine learning analysis of non-targeted metabolomics.”
Yosuke Hirakawa, Kentaro Yoshioka, Kensuke Kojima, Yasuho Yamashita, Takuma Shibahara, Takehiko Wada, Masaomi Nangaku, Reiko Inagi.
1

## Slide 2
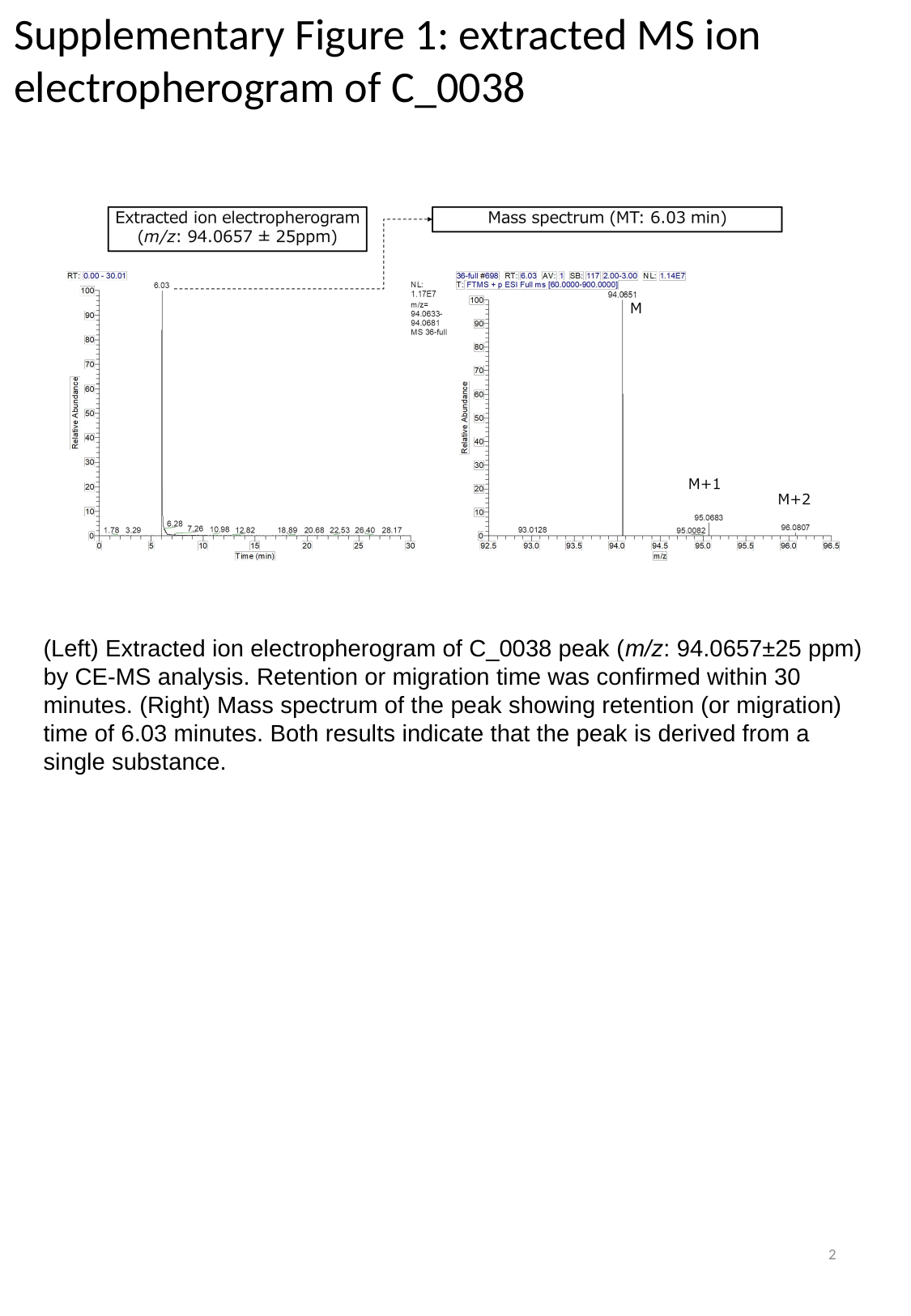

Supplementary Figure 1: extracted MS ion electropherogram of C_0038
(Left) Extracted ion electropherogram of C_0038 peak (m/z: 94.0657±25 ppm) by CE-MS analysis. Retention or migration time was confirmed within 30 minutes. (Right) Mass spectrum of the peak showing retention (or migration) time of 6.03 minutes. Both results indicate that the peak is derived from a single substance.
2

## Slide 3
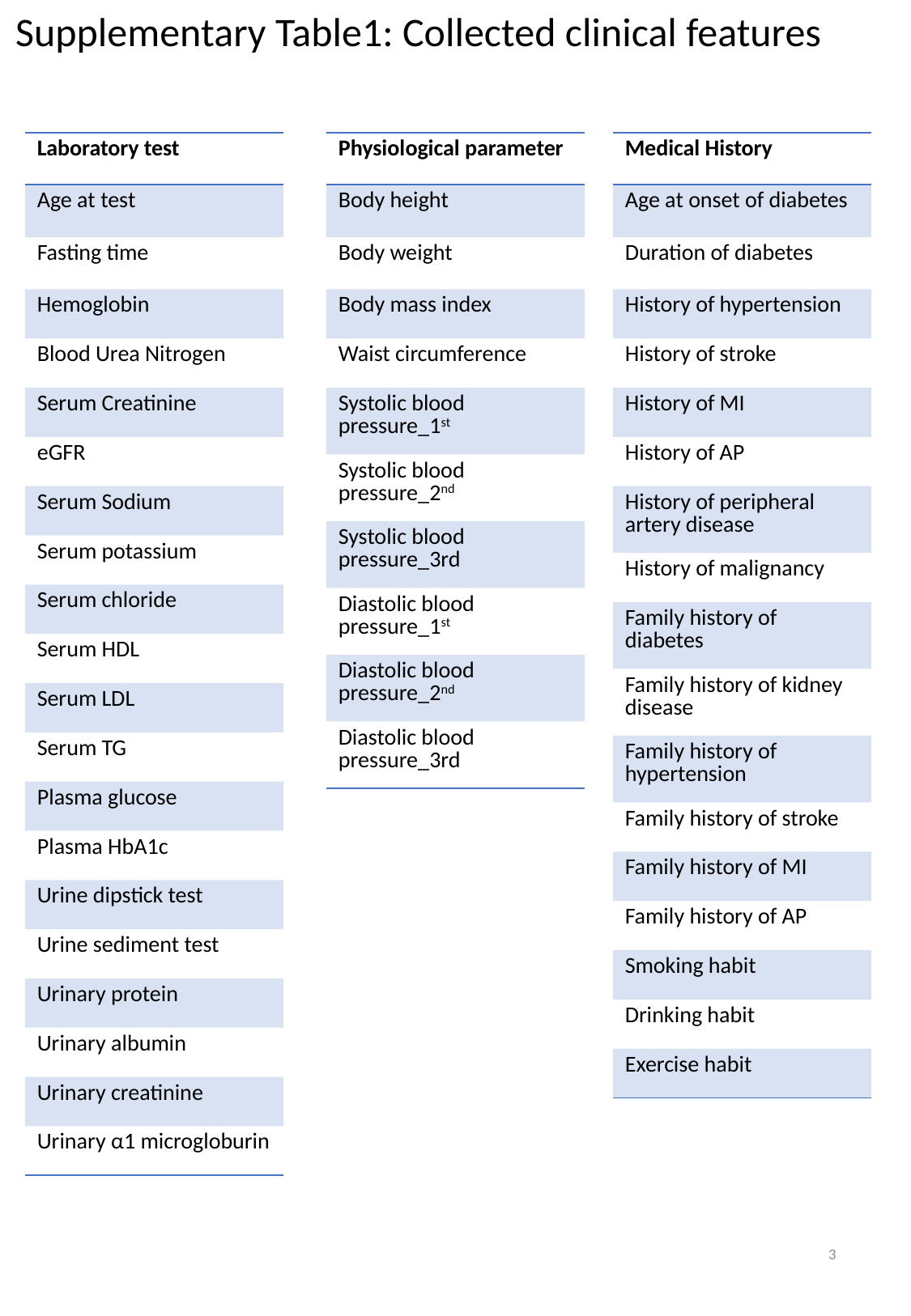

Supplementary Table1: Collected clinical features
| Laboratory test |
| --- |
| Age at test |
| Fasting time |
| Hemoglobin |
| Blood Urea Nitrogen |
| Serum Creatinine |
| eGFR |
| Serum Sodium |
| Serum potassium |
| Serum chloride |
| Serum HDL |
| Serum LDL |
| Serum TG |
| Plasma glucose |
| Plasma HbA1c |
| Urine dipstick test |
| Urine sediment test |
| Urinary protein |
| Urinary albumin |
| Urinary creatinine |
| Urinary α1 microgloburin |
| Physiological parameter |
| --- |
| Body height |
| Body weight |
| Body mass index |
| Waist circumference |
| Systolic blood pressure\_1st |
| Systolic blood pressure\_2nd |
| Systolic blood pressure\_3rd |
| Diastolic blood pressure\_1st |
| Diastolic blood pressure\_2nd |
| Diastolic blood pressure\_3rd |
| Medical History |
| --- |
| Age at onset of diabetes |
| Duration of diabetes |
| History of hypertension |
| History of stroke |
| History of MI |
| History of AP |
| History of peripheral artery disease |
| History of malignancy |
| Family history of diabetes |
| Family history of kidney disease |
| Family history of hypertension |
| Family history of stroke |
| Family history of MI |
| Family history of AP |
| Smoking habit |
| Drinking habit |
| Exercise habit |
3

## Slide 4
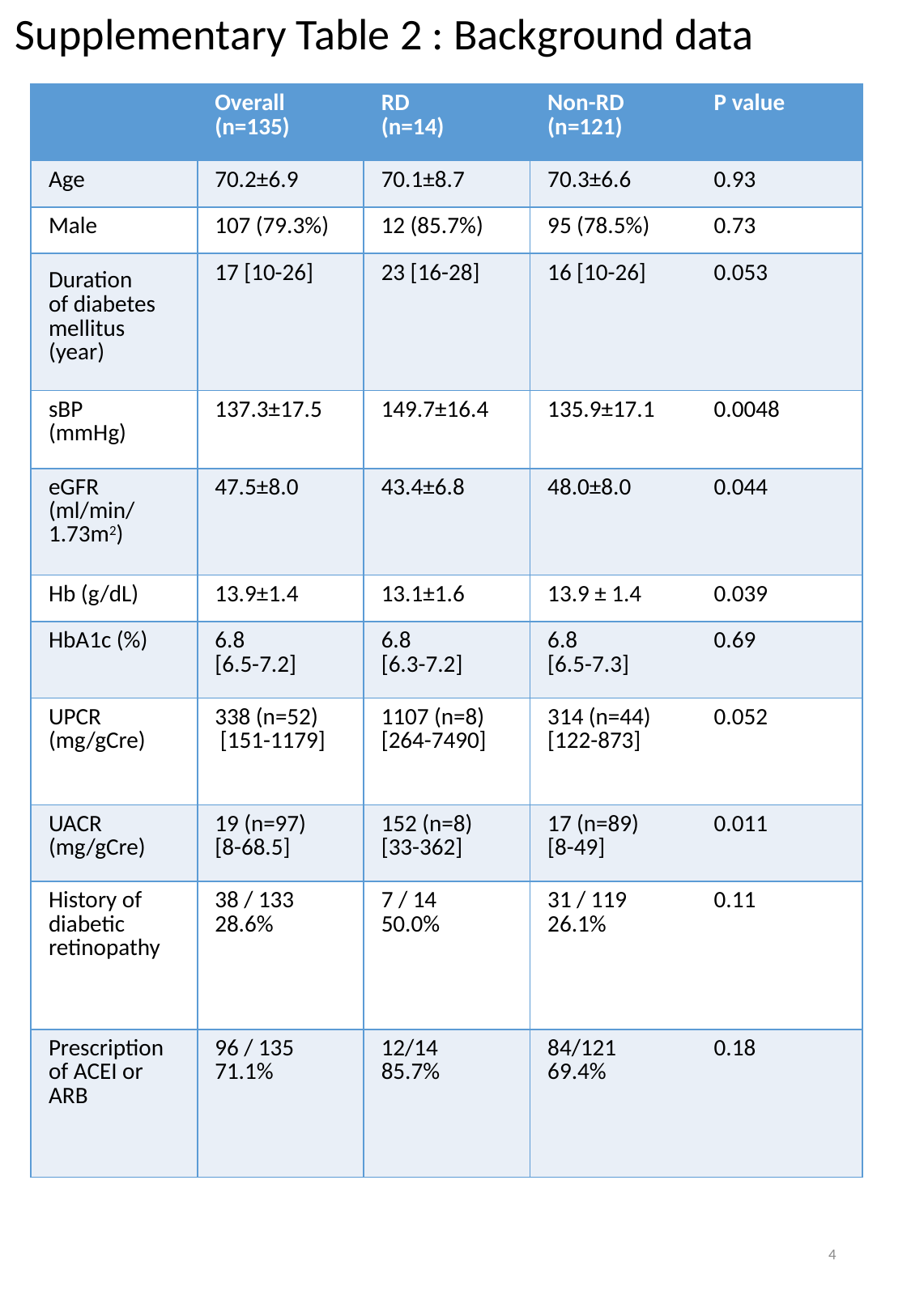

Supplementary Table 2 : Background data
| | Overall (n=135) | RD (n=14) | Non-RD (n=121) | P value |
| --- | --- | --- | --- | --- |
| Age | 70.2±6.9 | 70.1±8.7 | 70.3±6.6 | 0.93 |
| Male | 107 (79.3%) | 12 (85.7%) | 95 (78.5%) | 0.73 |
| Duration　of diabetes mellitus (year) | 17 [10-26] | 23 [16-28] | 16 [10-26] | 0.053 |
| sBP (mmHg) | 137.3±17.5 | 149.7±16.4 | 135.9±17.1 | 0.0048 |
| eGFR (ml/min/1.73m2) | 47.5±8.0 | 43.4±6.8 | 48.0±8.0 | 0.044 |
| Hb (g/dL) | 13.9±1.4 | 13.1±1.6 | 13.9 ± 1.4 | 0.039 |
| HbA1c (%) | 6.8 [6.5-7.2] | 6.8 [6.3-7.2] | 6.8 [6.5-7.3] | 0.69 |
| UPCR (mg/gCre) | 338 (n=52) [151-1179] | 1107 (n=8) [264-7490] | 314 (n=44) [122-873] | 0.052 |
| UACR (mg/gCre) | 19 (n=97) [8-68.5] | 152 (n=8) [33-362] | 17 (n=89) [8-49] | 0.011 |
| History of diabetic retinopathy | 38 / 133 28.6% | 7 / 14 50.0% | 31 / 119 26.1% | 0.11 |
| Prescription of ACEI or ARB | 96 / 135 71.1% | 12/14 85.7% | 84/121 69.4% | 0.18 |
4

## Slide 5
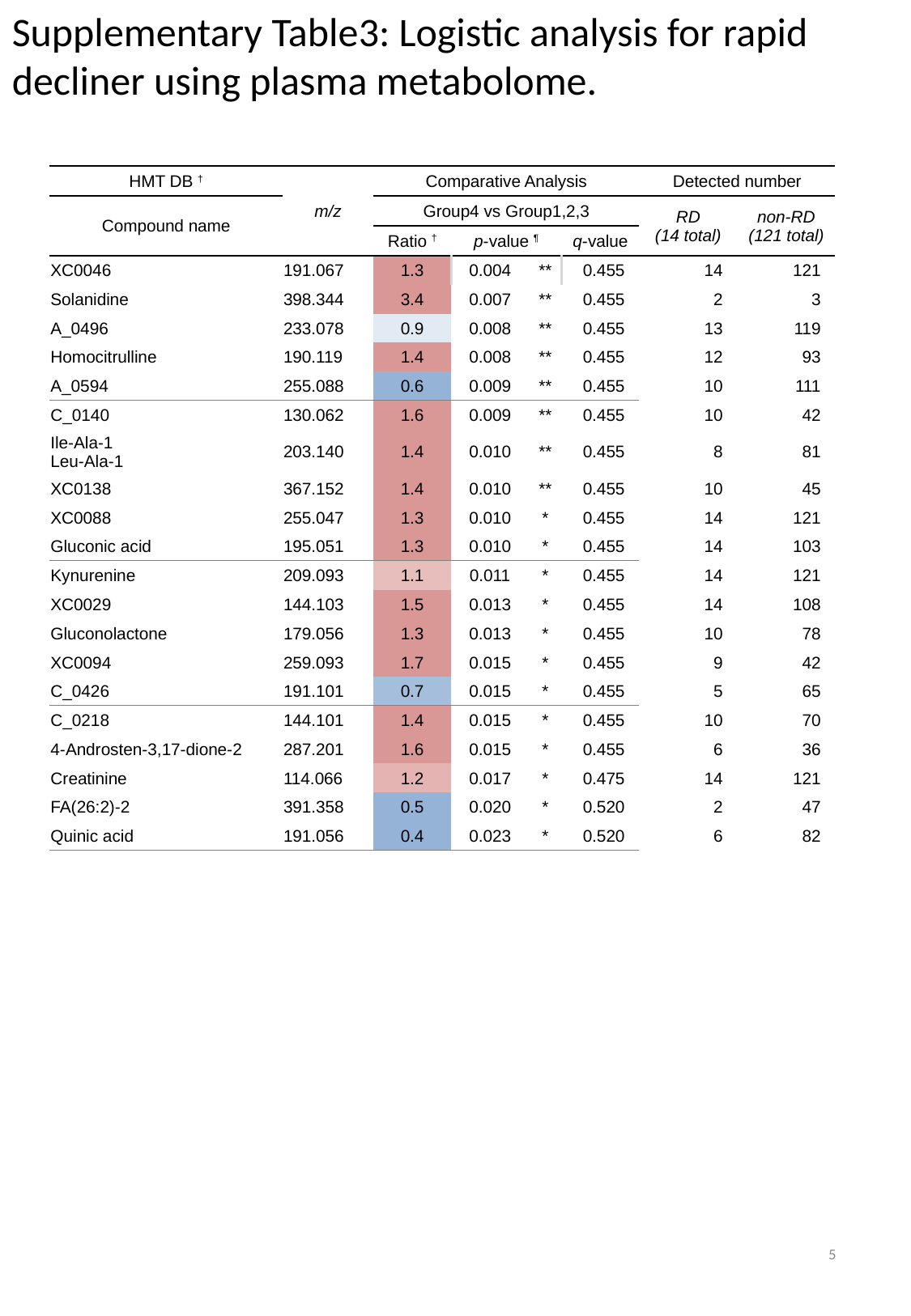

Supplementary Table3: Logistic analysis for rapid decliner using plasma metabolome.
| HMT DB † | m/z | Comparative Analysis | | | | Detected number | |
| --- | --- | --- | --- | --- | --- | --- | --- |
| Compound name | | Group4 vs Group1,2,3 | | | | RD (14 total) | non-RD (121 total) |
| | | Ratio † | p-value ¶ | | q-value | | |
| XC0046 | 191.067 | 1.3 | 0.004 | \*\* | 0.455 | 14 | 121 |
| Solanidine | 398.344 | 3.4 | 0.007 | \*\* | 0.455 | 2 | 3 |
| A\_0496 | 233.078 | 0.9 | 0.008 | \*\* | 0.455 | 13 | 119 |
| Homocitrulline | 190.119 | 1.4 | 0.008 | \*\* | 0.455 | 12 | 93 |
| A\_0594 | 255.088 | 0.6 | 0.009 | \*\* | 0.455 | 10 | 111 |
| C\_0140 | 130.062 | 1.6 | 0.009 | \*\* | 0.455 | 10 | 42 |
| Ile-Ala-1 Leu-Ala-1 | 203.140 | 1.4 | 0.010 | \*\* | 0.455 | 8 | 81 |
| XC0138 | 367.152 | 1.4 | 0.010 | \*\* | 0.455 | 10 | 45 |
| XC0088 | 255.047 | 1.3 | 0.010 | \* | 0.455 | 14 | 121 |
| Gluconic acid | 195.051 | 1.3 | 0.010 | \* | 0.455 | 14 | 103 |
| Kynurenine | 209.093 | 1.1 | 0.011 | \* | 0.455 | 14 | 121 |
| XC0029 | 144.103 | 1.5 | 0.013 | \* | 0.455 | 14 | 108 |
| Gluconolactone | 179.056 | 1.3 | 0.013 | \* | 0.455 | 10 | 78 |
| XC0094 | 259.093 | 1.7 | 0.015 | \* | 0.455 | 9 | 42 |
| C\_0426 | 191.101 | 0.7 | 0.015 | \* | 0.455 | 5 | 65 |
| C\_0218 | 144.101 | 1.4 | 0.015 | \* | 0.455 | 10 | 70 |
| 4-Androsten-3,17-dione-2 | 287.201 | 1.6 | 0.015 | \* | 0.455 | 6 | 36 |
| Creatinine | 114.066 | 1.2 | 0.017 | \* | 0.475 | 14 | 121 |
| FA(26:2)-2 | 391.358 | 0.5 | 0.020 | \* | 0.520 | 2 | 47 |
| Quinic acid | 191.056 | 0.4 | 0.023 | \* | 0.520 | 6 | 82 |
5

## Slide 6
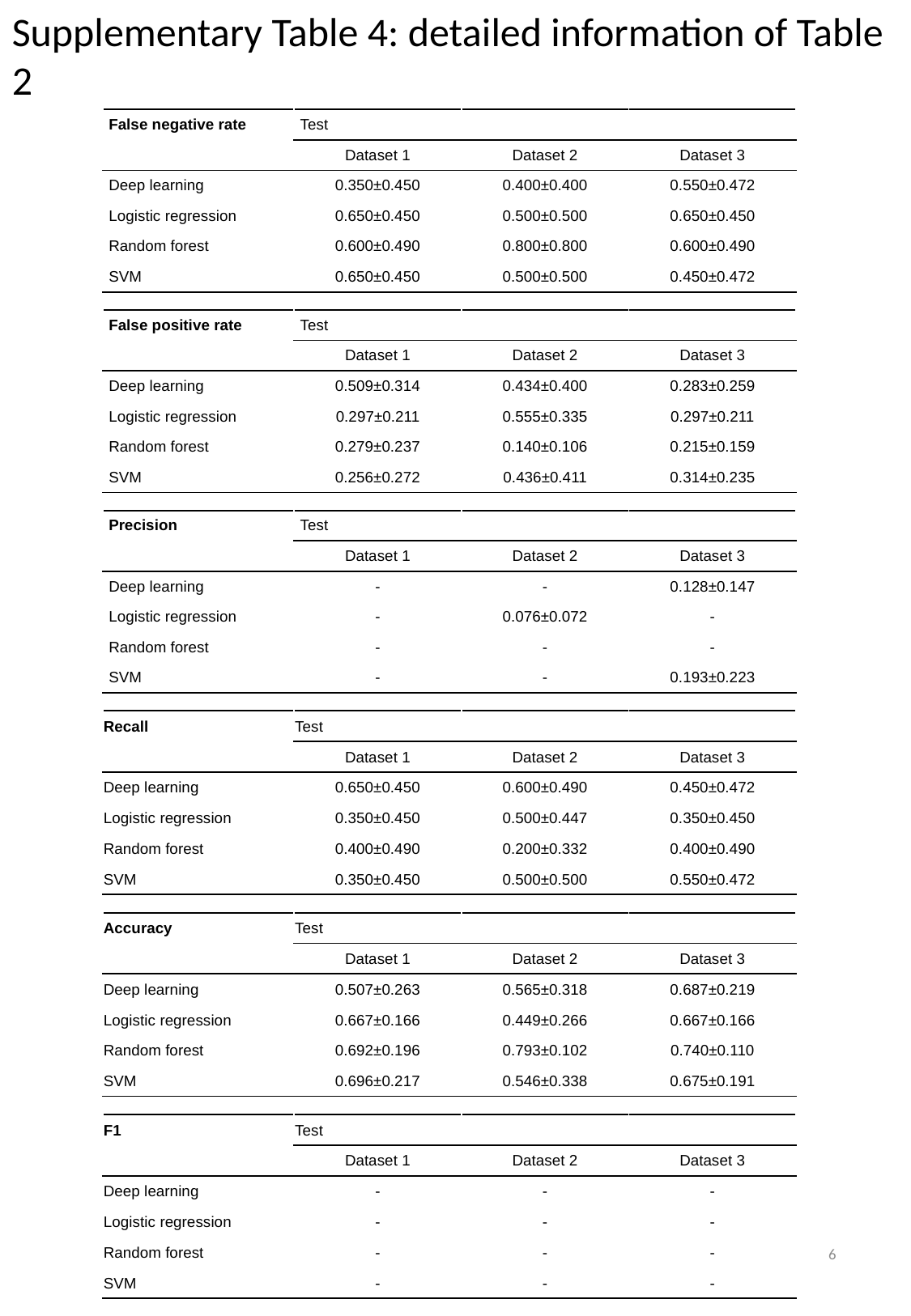

Supplementary Table 4: detailed information of Table 2
| False negative rate | Test | | |
| --- | --- | --- | --- |
| | Dataset 1 | Dataset 2 | Dataset 3 |
| Deep learning | 0.350±0.450 | 0.400±0.400 | 0.550±0.472 |
| Logistic regression | 0.650±0.450 | 0.500±0.500 | 0.650±0.450 |
| Random forest | 0.600±0.490 | 0.800±0.800 | 0.600±0.490 |
| SVM | 0.650±0.450 | 0.500±0.500 | 0.450±0.472 |
| False positive rate | Test | | |
| --- | --- | --- | --- |
| | Dataset 1 | Dataset 2 | Dataset 3 |
| Deep learning | 0.509±0.314 | 0.434±0.400 | 0.283±0.259 |
| Logistic regression | 0.297±0.211 | 0.555±0.335 | 0.297±0.211 |
| Random forest | 0.279±0.237 | 0.140±0.106 | 0.215±0.159 |
| SVM | 0.256±0.272 | 0.436±0.411 | 0.314±0.235 |
| Precision | Test | | |
| --- | --- | --- | --- |
| | Dataset 1 | Dataset 2 | Dataset 3 |
| Deep learning | - | - | 0.128±0.147 |
| Logistic regression | - | 0.076±0.072 | - |
| Random forest | - | - | - |
| SVM | - | - | 0.193±0.223 |
| Recall | Test | | |
| --- | --- | --- | --- |
| | Dataset 1 | Dataset 2 | Dataset 3 |
| Deep learning | 0.650±0.450 | 0.600±0.490 | 0.450±0.472 |
| Logistic regression | 0.350±0.450 | 0.500±0.447 | 0.350±0.450 |
| Random forest | 0.400±0.490 | 0.200±0.332 | 0.400±0.490 |
| SVM | 0.350±0.450 | 0.500±0.500 | 0.550±0.472 |
| Accuracy | Test | | |
| --- | --- | --- | --- |
| | Dataset 1 | Dataset 2 | Dataset 3 |
| Deep learning | 0.507±0.263 | 0.565±0.318 | 0.687±0.219 |
| Logistic regression | 0.667±0.166 | 0.449±0.266 | 0.667±0.166 |
| Random forest | 0.692±0.196 | 0.793±0.102 | 0.740±0.110 |
| SVM | 0.696±0.217 | 0.546±0.338 | 0.675±0.191 |
| F1 | Test | | |
| --- | --- | --- | --- |
| | Dataset 1 | Dataset 2 | Dataset 3 |
| Deep learning | - | - | - |
| Logistic regression | - | - | - |
| Random forest | - | - | - |
| SVM | - | - | - |
6

## Slide 7
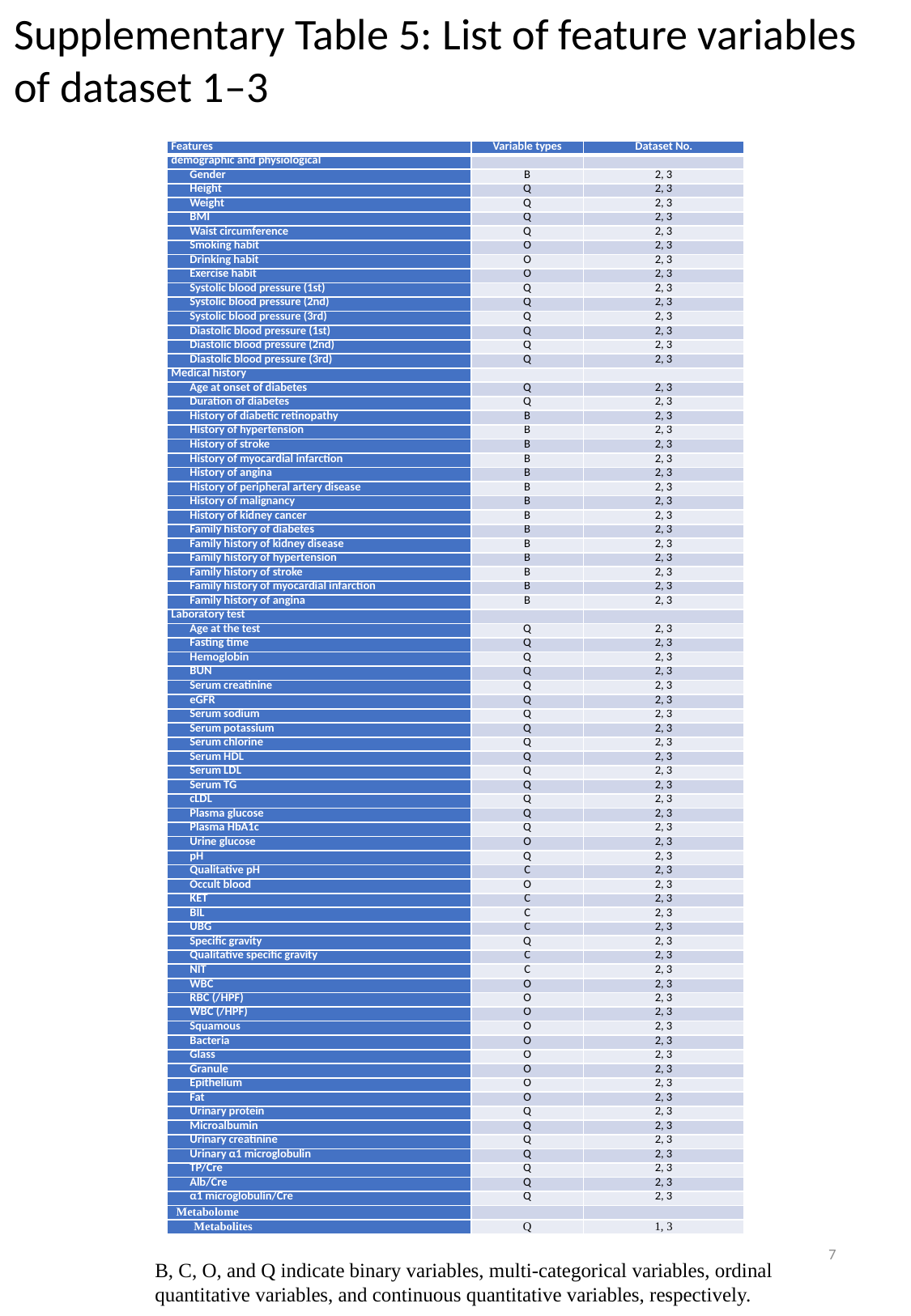

Supplementary Table 5: List of feature variables of dataset 1–3
| Features | Variable types | Dataset No. |
| --- | --- | --- |
| demographic and physiological | | |
| Gender | B | 2, 3 |
| Height | Q | 2, 3 |
| Weight | Q | 2, 3 |
| BMI | Q | 2, 3 |
| Waist circumference | Q | 2, 3 |
| Smoking habit | O | 2, 3 |
| Drinking habit | O | 2, 3 |
| Exercise habit | O | 2, 3 |
| Systolic blood pressure (1st) | Q | 2, 3 |
| Systolic blood pressure (2nd) | Q | 2, 3 |
| Systolic blood pressure (3rd) | Q | 2, 3 |
| Diastolic blood pressure (1st) | Q | 2, 3 |
| Diastolic blood pressure (2nd) | Q | 2, 3 |
| Diastolic blood pressure (3rd) | Q | 2, 3 |
| Medical history | | |
| Age at onset of diabetes | Q | 2, 3 |
| Duration of diabetes | Q | 2, 3 |
| History of diabetic retinopathy | B | 2, 3 |
| History of hypertension | B | 2, 3 |
| History of stroke | B | 2, 3 |
| History of myocardial infarction | B | 2, 3 |
| History of angina | B | 2, 3 |
| History of peripheral artery disease | B | 2, 3 |
| History of malignancy | B | 2, 3 |
| History of kidney cancer | B | 2, 3 |
| Family history of diabetes | B | 2, 3 |
| Family history of kidney disease | B | 2, 3 |
| Family history of hypertension | B | 2, 3 |
| Family history of stroke | B | 2, 3 |
| Family history of myocardial infarction | B | 2, 3 |
| Family history of angina | B | 2, 3 |
| Laboratory test | | |
| Age at the test | Q | 2, 3 |
| Fasting time | Q | 2, 3 |
| Hemoglobin | Q | 2, 3 |
| BUN | Q | 2, 3 |
| Serum creatinine | Q | 2, 3 |
| eGFR | Q | 2, 3 |
| Serum sodium | Q | 2, 3 |
| Serum potassium | Q | 2, 3 |
| Serum chlorine | Q | 2, 3 |
| Serum HDL | Q | 2, 3 |
| Serum LDL | Q | 2, 3 |
| Serum TG | Q | 2, 3 |
| cLDL | Q | 2, 3 |
| Plasma glucose | Q | 2, 3 |
| Plasma HbA1c | Q | 2, 3 |
| Urine glucose | O | 2, 3 |
| pH | Q | 2, 3 |
| Qualitative pH | C | 2, 3 |
| Occult blood | O | 2, 3 |
| KET | C | 2, 3 |
| BIL | C | 2, 3 |
| UBG | C | 2, 3 |
| Specific gravity | Q | 2, 3 |
| Qualitative specific gravity | C | 2, 3 |
| NIT | C | 2, 3 |
| WBC | O | 2, 3 |
| RBC (/HPF) | O | 2, 3 |
| WBC (/HPF) | O | 2, 3 |
| Squamous | O | 2, 3 |
| Bacteria | O | 2, 3 |
| Glass | O | 2, 3 |
| Granule | O | 2, 3 |
| Epithelium | O | 2, 3 |
| Fat | O | 2, 3 |
| Urinary protein | Q | 2, 3 |
| Microalbumin | Q | 2, 3 |
| Urinary creatinine | Q | 2, 3 |
| Urinary α1 microglobulin | Q | 2, 3 |
| TP/Cre | Q | 2, 3 |
| Alb/Cre | Q | 2, 3 |
| α1 microglobulin/Cre | Q | 2, 3 |
| Metabolome | | |
| Metabolites | Q | 1, 3 |
7
B, C, O, and Q indicate binary variables, multi-categorical variables, ordinal quantitative variables, and continuous quantitative variables, respectively.

## Slide 8
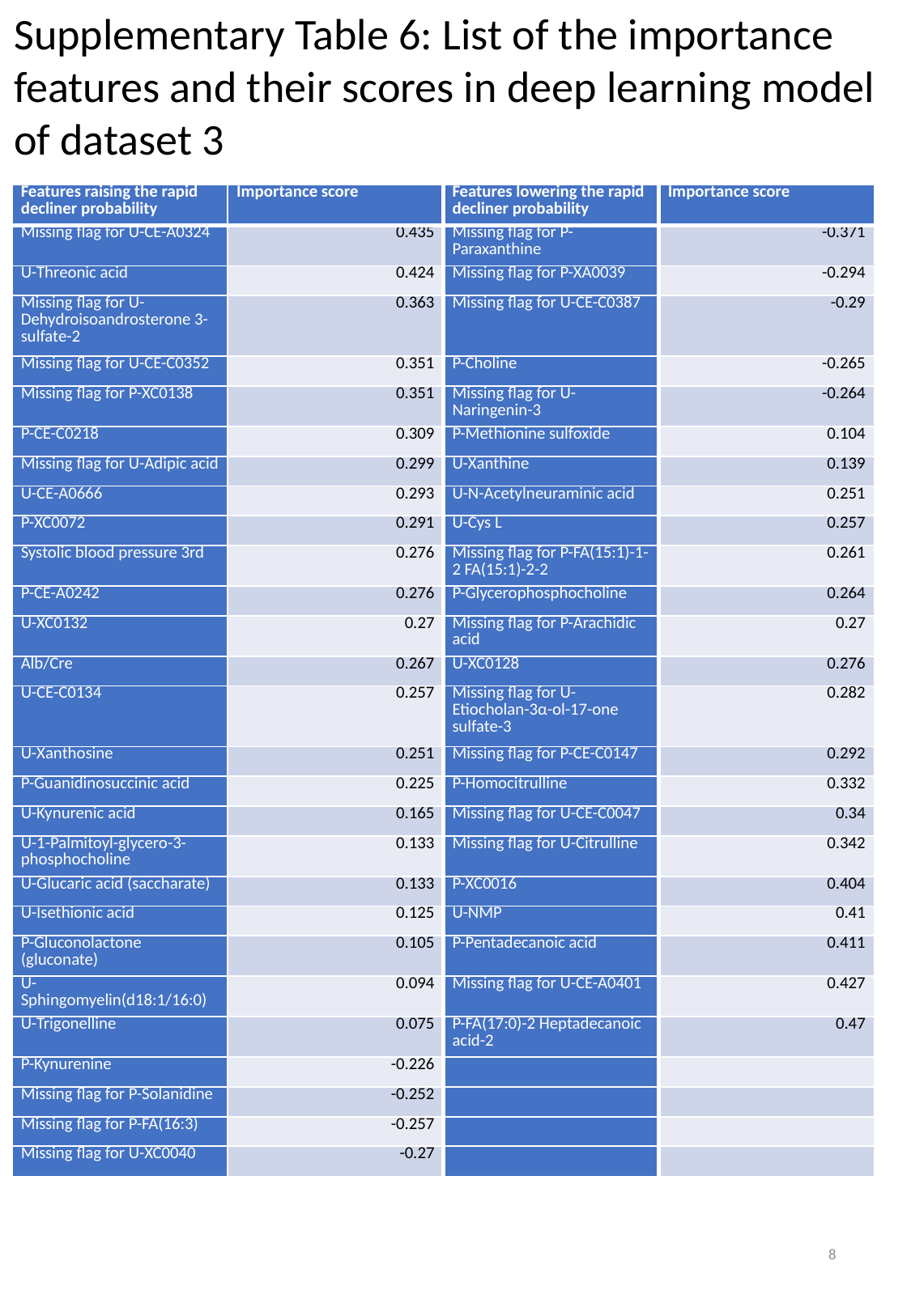

Supplementary Table 6: List of the importance
features and their scores in deep learning model of dataset 3
| Features raising the rapid decliner probability | Importance score | Features lowering the rapid decliner probability | Importance score |
| --- | --- | --- | --- |
| Missing flag for U-CE-A0324 | 0.435 | Missing flag for P-Paraxanthine | -0.371 |
| U-Threonic acid | 0.424 | Missing flag for P-XA0039 | -0.294 |
| Missing flag for U-Dehydroisoandrosterone 3-sulfate-2 | 0.363 | Missing flag for U-CE-C0387 | -0.29 |
| Missing flag for U-CE-C0352 | 0.351 | P-Choline | -0.265 |
| Missing flag for P-XC0138 | 0.351 | Missing flag for U-Naringenin-3 | -0.264 |
| P-CE-C0218 | 0.309 | P-Methionine sulfoxide | 0.104 |
| Missing flag for U-Adipic acid | 0.299 | U-Xanthine | 0.139 |
| U-CE-A0666 | 0.293 | U-N-Acetylneuraminic acid | 0.251 |
| P-XC0072 | 0.291 | U-Cys L | 0.257 |
| Systolic blood pressure 3rd | 0.276 | Missing flag for P-FA(15:1)-1-2 FA(15:1)-2-2 | 0.261 |
| P-CE-A0242 | 0.276 | P-Glycerophosphocholine | 0.264 |
| U-XC0132 | 0.27 | Missing flag for P-Arachidic acid | 0.27 |
| Alb/Cre | 0.267 | U-XC0128 | 0.276 |
| U-CE-C0134 | 0.257 | Missing flag for U-Etiocholan-3α-ol-17-one sulfate-3 | 0.282 |
| U-Xanthosine | 0.251 | Missing flag for P-CE-C0147 | 0.292 |
| P-Guanidinosuccinic acid | 0.225 | P-Homocitrulline | 0.332 |
| U-Kynurenic acid | 0.165 | Missing flag for U-CE-C0047 | 0.34 |
| U-1-Palmitoyl-glycero-3-phosphocholine | 0.133 | Missing flag for U-Citrulline | 0.342 |
| U-Glucaric acid (saccharate) | 0.133 | P-XC0016 | 0.404 |
| U-Isethionic acid | 0.125 | U-NMP | 0.41 |
| P-Gluconolactone (gluconate) | 0.105 | P-Pentadecanoic acid | 0.411 |
| U-Sphingomyelin(d18:1/16:0) | 0.094 | Missing flag for U-CE-A0401 | 0.427 |
| U-Trigonelline | 0.075 | P-FA(17:0)-2 Heptadecanoic acid-2 | 0.47 |
| P-Kynurenine | -0.226 | | |
| Missing flag for P-Solanidine | -0.252 | | |
| Missing flag for P-FA(16:3) | -0.257 | | |
| Missing flag for U-XC0040 | -0.27 | | |
8

## Slide 9
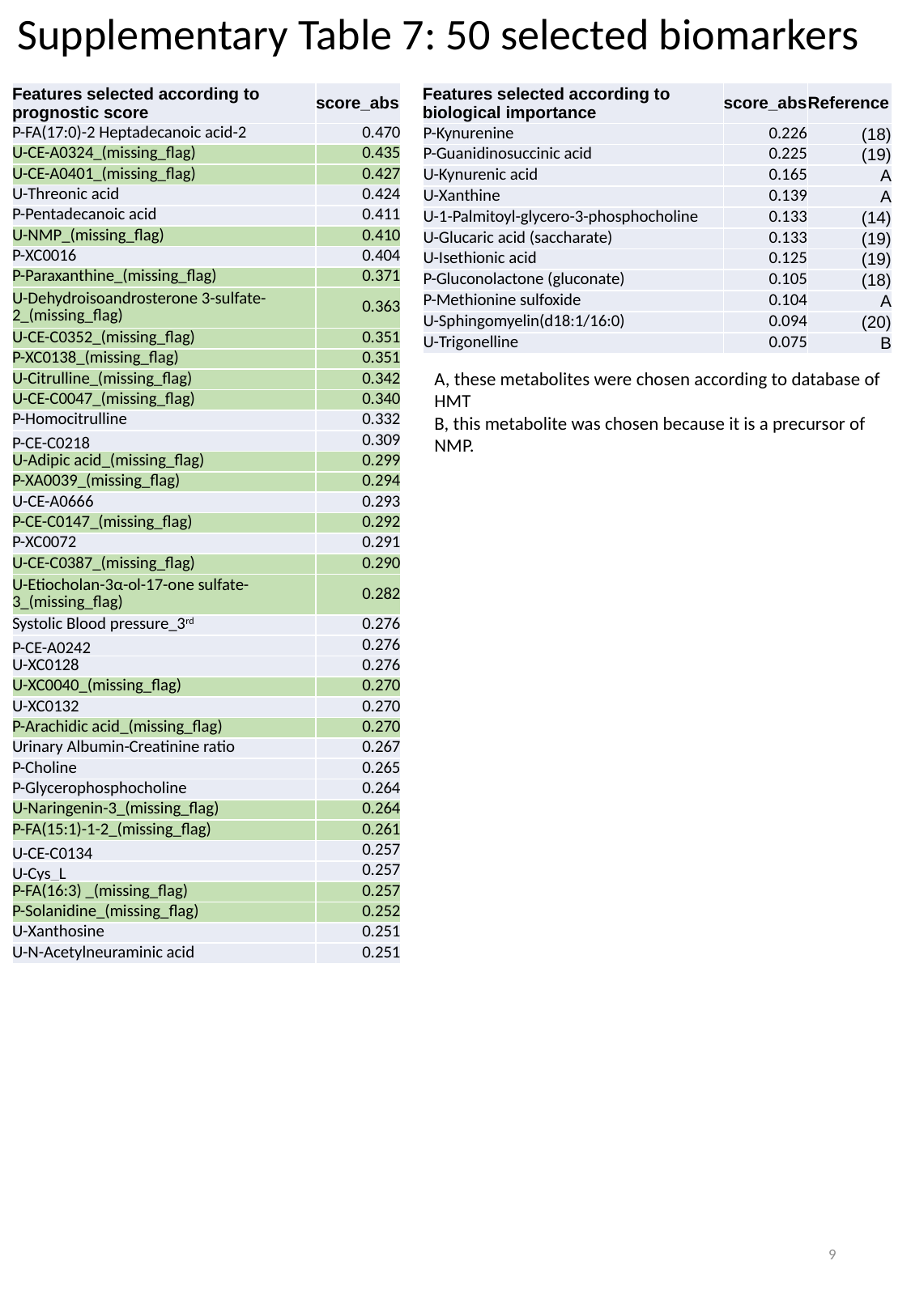

Supplementary Table 7: 50 selected biomarkers
| Features selected according to prognostic score | score\_abs |
| --- | --- |
| P-FA(17:0)-2 Heptadecanoic acid-2 | 0.470 |
| U-CE-A0324\_(missing\_flag) | 0.435 |
| U-CE-A0401\_(missing\_flag) | 0.427 |
| U-Threonic acid | 0.424 |
| P-Pentadecanoic acid | 0.411 |
| U-NMP\_(missing\_flag) | 0.410 |
| P-XC0016 | 0.404 |
| P-Paraxanthine\_(missing\_flag) | 0.371 |
| U-Dehydroisoandrosterone 3-sulfate-2\_(missing\_flag) | 0.363 |
| U-CE-C0352\_(missing\_flag) | 0.351 |
| P-XC0138\_(missing\_flag) | 0.351 |
| U-Citrulline\_(missing\_flag) | 0.342 |
| U-CE-C0047\_(missing\_flag) | 0.340 |
| P-Homocitrulline | 0.332 |
| P-CE-C0218 | 0.309 |
| U-Adipic acid\_(missing\_flag) | 0.299 |
| P-XA0039\_(missing\_flag) | 0.294 |
| U-CE-A0666 | 0.293 |
| P-CE-C0147\_(missing\_flag) | 0.292 |
| P-XC0072 | 0.291 |
| U-CE-C0387\_(missing\_flag) | 0.290 |
| U-Etiocholan-3α-ol-17-one sulfate-3\_(missing\_flag) | 0.282 |
| Systolic Blood pressure\_3rd | 0.276 |
| P-CE-A0242 | 0.276 |
| U-XC0128 | 0.276 |
| U-XC0040\_(missing\_flag) | 0.270 |
| U-XC0132 | 0.270 |
| P-Arachidic acid\_(missing\_flag) | 0.270 |
| Urinary Albumin-Creatinine ratio | 0.267 |
| P-Choline | 0.265 |
| P-Glycerophosphocholine | 0.264 |
| U-Naringenin-3\_(missing\_flag) | 0.264 |
| P-FA(15:1)-1-2\_(missing\_flag) | 0.261 |
| U-CE-C0134 | 0.257 |
| U-Cys\_L | 0.257 |
| P-FA(16:3) \_(missing\_flag) | 0.257 |
| P-Solanidine\_(missing\_flag) | 0.252 |
| U-Xanthosine | 0.251 |
| U-N-Acetylneuraminic acid | 0.251 |
| Features selected according to biological importance | score\_abs | Reference |
| --- | --- | --- |
| P-Kynurenine | 0.226 | (18) |
| P-Guanidinosuccinic acid | 0.225 | (19) |
| U-Kynurenic acid | 0.165 | A |
| U-Xanthine | 0.139 | A |
| U-1-Palmitoyl-glycero-3-phosphocholine | 0.133 | (14) |
| U-Glucaric acid (saccharate) | 0.133 | (19) |
| U-Isethionic acid | 0.125 | (19) |
| P-Gluconolactone (gluconate) | 0.105 | (18) |
| P-Methionine sulfoxide | 0.104 | A |
| U-Sphingomyelin(d18:1/16:0) | 0.094 | (20) |
| U-Trigonelline | 0.075 | B |
A, these metabolites were chosen according to database of HMT
B, this metabolite was chosen because it is a precursor of NMP.
9

## Slide 10
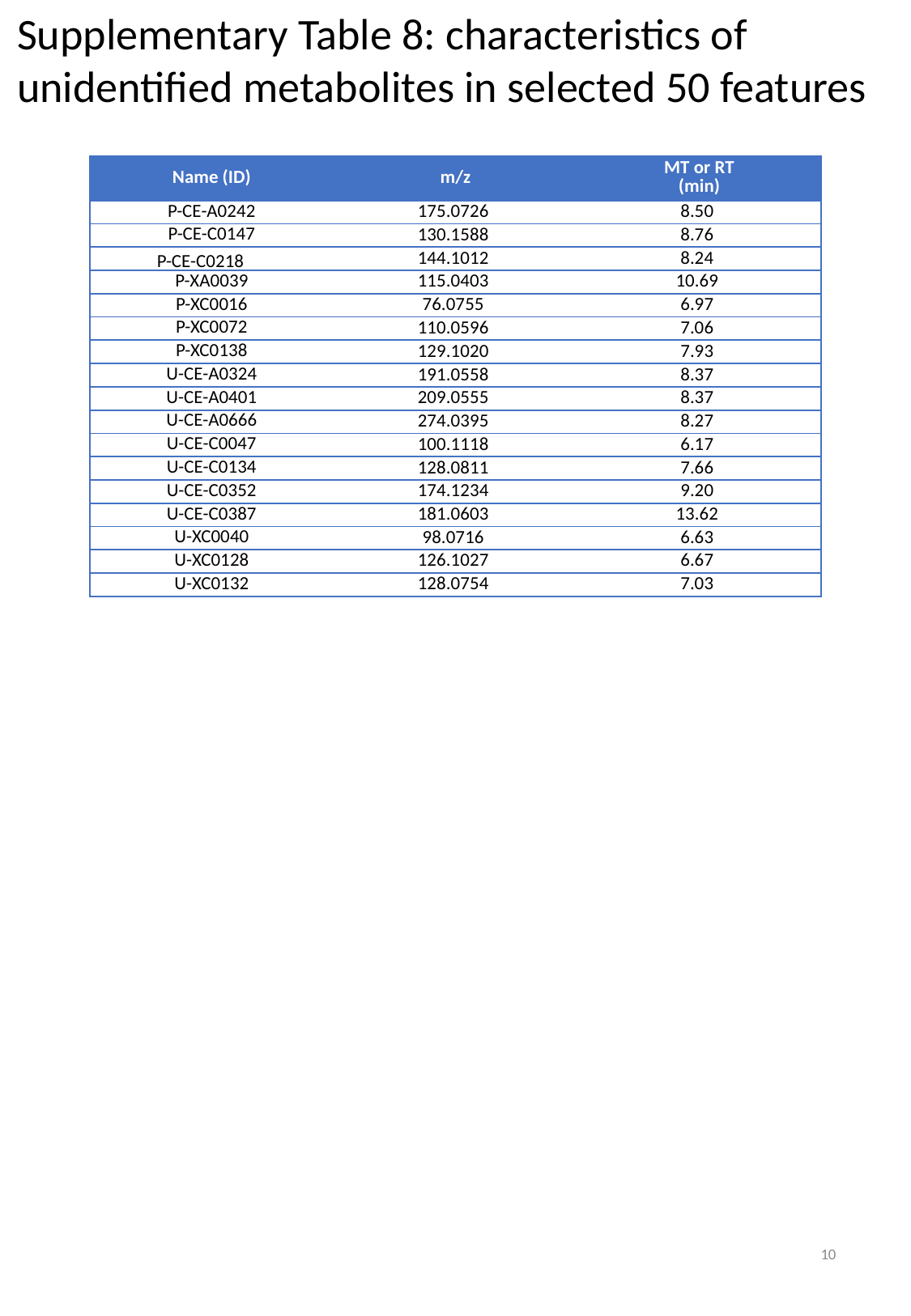

Supplementary Table 8: characteristics of
unidentified metabolites in selected 50 features
| Name (ID) | m/z | MT or RT (min) |
| --- | --- | --- |
| P-CE-A0242 | 175.0726 | 8.50 |
| P-CE-C0147 | 130.1588 | 8.76 |
| P-CE-C0218 | 144.1012 | 8.24 |
| P-XA0039 | 115.0403 | 10.69 |
| P-XC0016 | 76.0755 | 6.97 |
| P-XC0072 | 110.0596 | 7.06 |
| P-XC0138 | 129.1020 | 7.93 |
| U-CE-A0324 | 191.0558 | 8.37 |
| U-CE-A0401 | 209.0555 | 8.37 |
| U-CE-A0666 | 274.0395 | 8.27 |
| U-CE-C0047 | 100.1118 | 6.17 |
| U-CE-C0134 | 128.0811 | 7.66 |
| U-CE-C0352 | 174.1234 | 9.20 |
| U-CE-C0387 | 181.0603 | 13.62 |
| U-XC0040 | 98.0716 | 6.63 |
| U-XC0128 | 126.1027 | 6.67 |
| U-XC0132 | 128.0754 | 7.03 |
10
